# Modeling musculoskeletal kinematic and dynamic redundancy using null space projection

**DOI:** 10.1371/journal.pone.0209171

**Published:** 2019-01-02

**Authors:** Dimitar Stanev, Konstantinos Moustakas

**Affiliations:** Department of Electrical and Computer Engineering, University of Patras, Patras, Achaia, Greece; University of Münster, GERMANY

## Abstract

The coordination of the human musculoskeletal system is deeply influenced by its redundant structure, in both kinematic and dynamic terms. Noticing a lack of a relevant, thorough treatment in the literature, we formally address the issue in order to understand and quantify factors affecting the motor coordination. We employed well-established techniques from linear algebra and projection operators to extend the underlying kinematic and dynamic relations by modeling the redundancy effects in null space. We distinguish three types of operational spaces, namely task, joint and muscle space, which are directly associated with the physiological factors of the system. A method for consistently quantifying the redundancy on multiple levels in the entire space of feasible solutions is also presented. We evaluate the proposed muscle space projection on segmental level reflexes and the computation of the feasible muscle forces for arbitrary movements. The former proves to be a convenient representation for interfacing with segmental level models or implementing controllers for tendon driven robots, while the latter enables the identification of force variability and correlations between muscle groups, attributed to the system’s redundancy. Furthermore, the usefulness of the proposed framework is demonstrated in the context of estimating the bounds of the joint reaction loads, where we show that misinterpretation of the results is possible if the null space forces are ignored. This work presents a theoretical analysis of the redundancy problem, facilitating application in a broad range of fields related to motor coordination, as it provides the groundwork for null space characterization. The proposed framework rigorously accounts for the effects of kinematic and dynamic redundancy, incorporating it directly into the underlying equations using the notion of null space projection, leading to a complete description of the system.

## Introduction

The human musculoskeletal system has a redundant structure, i.e., there are more Degrees of Freedom (DoFs) than those required to perform a certain task (kinematic redundancy) and each DoF is actuated by multiple muscles (dynamic redundancy). This over-availability poses numerous challenges in the process of modeling and simulation that can negatively affect the validity of the models and the obtained results, rendering their application frequently inappropriate for clinical practice. Despite the fact that mathematical tools for studying redundant systems do exist, they are still not widely adopted in the field of musculoskeletal simulation. Proper formalization and interpretation of redundancy can significantly improve our understanding of the motor coordination problem.

We introduce an extended derivation of the underlying equations that govern the kinematic and dynamic evolution of the system, accounting explicitly for the different types of redundancy presented. The derivations rely on the theory of linear projection operators and the properties of the associated subspaces. An intuitive analogy is the projection of a point from 3D space to a point on a plane (2D). When mapping from a high-dimensional subspace the problem is straightforward, however, the projection from 2D to 3D is not unique. By incorporating the null space one can identify the feasible solution space and outline the factors that influence it.

What makes musculoskeletal systems challenging is not only the process of creating accurate mathematical representations, but also the assumptions that are introduced when they are analyzed. Traditional solutions of the muscle redundancy problem [1], such as minimum effort criterion, are very popular and have roots in a compelling evolutionary hypothesis, which states that motor control systems evolve to minimize energy expenditure during movement. Undeniably, single solution methods are of great importance, however, the assumptions that are introduced can severely hinder the validity of the obtained results. This is especially evident in the case of rigidity [2] in Parkinson’s disease, which is characterized by the inability of the muscles to relax. Clearly, a particular solution will not only bias the results, but will also affect the calculation of other quantities that depend on the muscle forces, like joint reaction loads. On the other hand, identification of the feasible solution space, as outlined in this work, can help to properly interpret results obtained from the redundant musculoskeletal systems.

### Contribution—Motivation

The main contributions of this work can be summarized below:

- Identify the properties of kinematic redundancy when performing a specific task and establish the kinematic and dynamic relations (sections “Relation between task and joint space quantities” and “Task space equations of motion”). Determine the properties of dynamic redundancy and their relationship to joint and muscle space quantities both at a kinematic and dynamic level (sections “Relation between muscle and joint space quantities” and “Muscle space equations of motion”). This description can potentially enable the modeling and evaluation of hypotheses related to normal and pathological conditions presented in the coordination process (e.g., slack muscle disorder, increase in joint stiffness, rigidity, etc.). We also show that these disorders manifest themselves in the null space.
- Propose an alternative representation of the Equations of Motion (EoMs) in muscle space (section “Muscle space equations of motion”). Muscle space projection is a convenient representation for interfacing with segmental level models (e.g., reflexes) [3] or implementing controllers for tendon driven robots [4]. Furthermore, it has the highest number of DoFs and permits coordination of different aspects of the movement (e.g., co-contraction) that are uncontrollable in subspaces of lower dimensionality. Task and joint space projections are specializations of muscle space.
- Establish an effective model for quantifying redundancy with respect to the movement task, the neuromuscular model (e.g., linear/nonlinear muscle model, synergy encoding [5]) and the anatomical characteristics of the muscle routing (the muscle moment arm and its null space) (section “Exploitation of kinematic and dynamic redundancy”). This approach is advantageous as compared to the local techniques (e.g., minimum effort optimization [1]) that are suitable for finding only a particular solution, because it identifies the entire solution space of possible muscle force realizations as well as the factors that influence its structure. The importance of identifying the entire solution space when estimating the bounds of the joint reaction loads is demonstrated and we further show that misinterpretation of the results is possible if local techniques are used as compared to the proposed approach.

The results demonstrate working examples of the developed models. The projected EoMs in muscle space provide a new perspective for studying the musculoskeletal system in this domain. In section “Muscle space projection and reflexes”, we design a posture controller that encapsulates the characteristics of the internal regulation process performed in the spinal cord and study the response of the system under external disturbances. Muscle space projection proves to be a convenient representation to solve this problem, since no conversion to muscle activation is required.

In section “Characterization of the feasible muscle force space”, we present a case study in which we determine the force variability and correlations between different muscle groups for arbitrary movements and show that there is a significant systematic correlation between different muscle pairs that emerges from the muscle routing properties and the functional constraints of the task. This can be used to estimate the importance of actors/muscles during the movement, to identify synergies and classify the various control strategies available [6, 7], considering the entire solution space. This not only demonstrates an effective approach for finding the family of possible solutions, but also exposes the structural relations of the null space solutions.

In section “The effect of null space on the joint reaction loads”, we perform a joint reaction analysis on a gait, using a realistic full body model, appropriately accounting for the null space muscle forces that do not alter the movement. Accurate joint reaction force estimation requires reliable assessment of the muscle forces. As this is an impossible task, we demonstrate the usefulness of identifying the feasible muscle forces in order to determine the limits of the reaction loads. The large variance that is evident from the results confirms that misinterpretation of the reaction loads is possible if the null space contribution is ignored.

To the best of our knowledge this is the first study that incorporates the null space as shaped by the redundant nature of the system into the kinematic and dynamic relations. We investigate the properties of these projections with emphasis on their physiological counterparts. Thus, a complete theoretical analysis is constructed, which can be extended for many practical applications related to the motor coordination. The source code along with any related material for this publication are publicly available, providing simple examples so that the readers can reproduce, understand and reuse the presented methods (S1 File).

### Related work

Motor control is the process by which humans and animals use their nervous system to activate and coordinate the muscles and limbs involved in the performance of a movement. The redundant nature of the musculoskeletal system was noted over fifty years ago [8], highlighting the over-availability of DoFs for most common tasks. Subsequently, researchers have created formal descriptions of the physiological process in order to identify the main variables responsible for coordinated movement [9–12]. Important hypotheses relative to these have been developed, improving our understanding of the central coordination problem [13, 14].

It has been shown that the musculoskeletal system can act as a “filter” that transforms the feasible muscle activations into a set of feasible wrenches [15, 16], revealing an important direction towards a holistic identification of the system capabilities. We propose a more general approach (section “Exploitation of kinematic and dynamic redundancy”), where the computed feasible muscle forces are both action-specific, accounting for the dynamic evolution of the motion, and satisfy the physiological constraints of the muscles, outlining the various factors that affect the solution space. Since the entire solution space is obtained using our framework, we can quantifiably explore the available strategies to the Central Nervous System (CNS). This is useful because it is still unclear how the CNS explores the musculoskeletal redundancy by controlling a low-dimensional subset of synergies [5, 17] and whether there is a correlation with the structure of the musculoskeletal system and the functional constraints each task [16, 18]. We will show that both the structure of the model and the task (movement) indirectly impose some degree of muscle correlation (section “Characterization of the feasible muscle force space”).

The use of linear projection operators have been successfully utilized in the field of robotics [19–21] in order to separate and simplify the control problem into multiple objectives. It has been shown that task-based projection can be used effectively for planning and simulation of constrained musculoskeletal systems [22, 23] and furthermore, it provides the means to identify the kinematically redundant DoFs. In a similar vein, we explore the null space properties as well as the potential applications that exploit not only the kinematic redundancy, but also the dynamic redundancy of the system. Detailed derivations are presented in order to provide the reader with the appropriate background to understand the implications of the proposed models for addressing redundant systems.

## Preliminaries—Notation

The mathematical notation follows the ISO guidelines. Variables are set in italic (e.g., $a\in R^{}$); constants and function are set in roman; vector and matrices are set in bold italic. Vectors are denoted by lower-case letters (e.g., $c\in R^{m}$) and matrices by upper-case (e.g., $C\in R^{n\times n}$).

Linear projection operators are used extensively, deeming the introduction of the relevant notation necessary. For a given linear transformation

$$\begin{array}{c} Ax=b,\;x\in {ℜ}^{n},\;b\in {ℜ}^{m}\\ A=\left[\begin{array}{ccc} a_{1,1} & \cdots & a_{1,n}\\ \vdots & \ddots & \vdots \\ a_{m,1} & \cdots & a_{m,n} \end{array}\right]=\left[\begin{array}{ccc} | & & |\\ c_{1} & \cdots & c_{n}\\ | & & | \end{array}\right]=\left[\begin{array}{ccc} - & r_{1}^{T} & -\\ & \vdots & \\ - & r_{m}^{T} & - \end{array}\right] \end{array}$$ (1)

where the ***A*** matrix defines a mapping $A:R^{n}\to R^{m}$. The column space (a.k.a. image or range) of ***A*** is a space spanned by its *n* *m*-dimensional column vectors

$$\begin{array}{c} C\left(A\right)=span\left(c_{1},\;\ldots ,\;c_{n}\right)\subseteq R^{m} \end{array}$$ (2)

which is an *r*-dimensional (*r* ≤ *n* independent columns) subspace of $R^{m}$ composed of all possible linear combinations of its *n* column vectors. The row space of ***A*** is a space spanned by its *m* *n*-dimensional row vectors

$$\begin{array}{c} R\left(A\right)=span\left(r_{1},\;\ldots ,\;r_{m}\right)\subseteq R^{n} \end{array}$$ (3)

which is an *r*-dimensional subspace of $R^{n}$ composed of all possible linear combinations of its *m* row vectors. The left null space of ***A*** (N(A)), is the set of all ***x*** that satisfy the homogeneous equation

$$\begin{array}{c} N\left(A\right)=\left\{x\in R^{n}:Ax=0\right\}\subseteq R^{n} \end{array}$$ (4)

and similarly the right null space of ***A*** ($N(A^{T})$) is the set of all ***b*** that satisfy

$$\begin{array}{c} N\left(A^{T}\right)=\left\{b\in R^{m}:A^{T}b=0\right\}\subseteq R^{m}. \end{array}$$ (5)

The following properties between the various subspaces hold for an operator ***A*** (⊥ stand for orthogonal complement and the symbol ⊕ denotes the direct sum)

$$\begin{array}{c} \mathbb{R}(A)=\mathbb{C}\left(A^{T}\right),\mathbb{C}(A)=\mathbb{R}\left(A^{T}\right)\\ \mathbb{R}(A)\cap \mathbb{N}(A)=\emptyset ,\mathbb{R}(A)⊥\mathbb{N}(A),\mathbb{R}(A)⊕\mathbb{N}(A)={ℜ}^{n}\\ \mathbb{C}(A)\cap \mathbb{N}\left(A^{T}\right)=\emptyset ,\mathbb{C}(A)⊥\mathbb{N}\left(A^{T}\right),\mathbb{C}(A)⊕\mathbb{N}\left(A^{T}\right)={ℜ}^{m}. \end{array}$$ (6)

**Fig 1:**
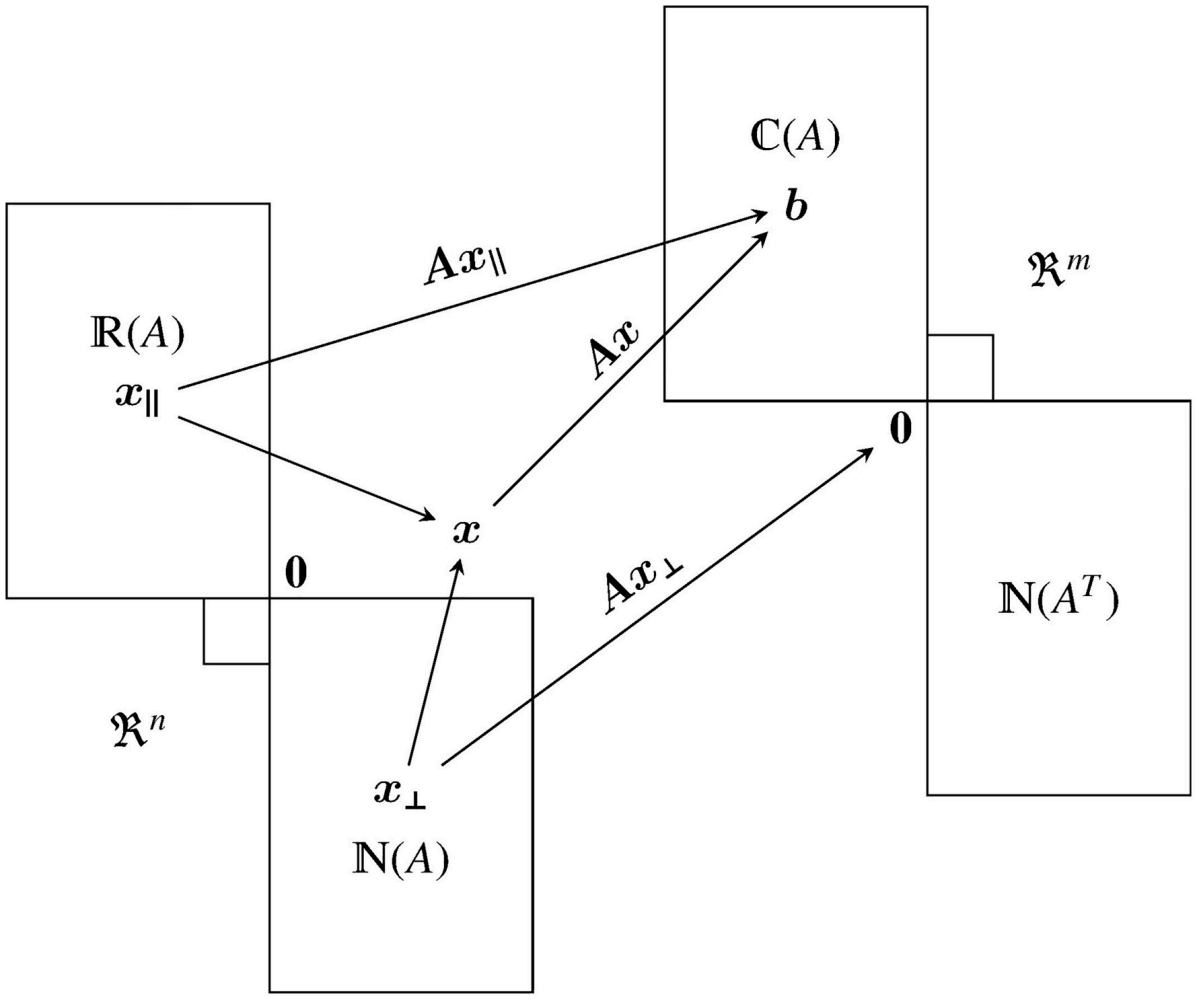
Subspace projection. Various relations between the different subspaces associated with matrix ***A***. Note that the null space solutions have zero contribution when projected by ***A***.

The general solution of Eq (1) in the underdetermined case (*n* > *m*), since this is how redundancy is presented, is of the form

$$\begin{array}{c} x=x_{∥}+x_{⊥} \end{array}$$ (7)

where $x_{∥}\in R\left(A\right)$ is a particular solution and $x_{⊥}\in N\left(A\right)$ is an arbitrary vector that belongs to null space of ***A*** (***Ax***_⊥_ = **0**). The various relations between the different subspaces can be summarized in Fig 1.

A projection operator ***P*** is a linear transformation from a vector space to itself, for which the idempotence property holds (***P***^2^ = ***P***). The operator is termed orthogonal if ***P*** = ***P***^*T*^ [20], which is equivalent to the range and the null space being mutually orthogonal. We continue by defining four projection operators, their properties and relations among the different subspaces for a general system ***Ax*** = ***b***.

**Defination 1**. *[Projection operators]*

*Let **T**_**A**_ = **AA**^+^ (**T** is used if **A** is a tall matrix) and **F_A_** = **A**^+^**A** (**F** is used if **A** is a fat matrix) be two orthogonal projection operators derived from matrix **A**, where **A**^+^ is the Moore-Penrose Pseudoinverse (MPP) matrix. The following assertions hold true*:

1. $T_{A}^{T}=T_{A}$ *and* $T_{A}^{2}=T_{A}$.
2. $F_{A}^{T}=F_{A}$ *and* $F_{A}^{2}=F_{A}$.
3. ***T**_**A**_**A** = **AF**_**A**_ = **A** and **A**^+^**T**_**A**_ = **F**_**A**_**A**^+^ = **A**^+^*.
4. ***T**_**A**_ is the projector onto the* C(A).
5. ***F**_**A**_ is the projector onto the* R(A).
6. $N_{T_{A}}=\left(I-T_{A}\right)$ *is the projector onto the* $N(A^{T})$.
7. $N_{F_{A}}=\left(I-F_{A}\right)$ *is the projector onto the* N(A).
8. $N_{T_{A}}A=0$ *and* $AN_{F_{A}}=0$ *are the left and right null spaces, respectively*.

*Proof*. Appendix “Projection operators”.

The following definition summarizes some useful properties of the MPP matrix which is used extensively in this work.

**Definition 2**. *[Moore—Penrose pseudoinverse]*

*For a matrix* $A\in R^{m\times n}$, *the MPP of **A** is defined as the matrix* $A^{+}\in R^{n\times m}$, *satisfying all the following*:

1. ***AA***^+^***A*** = ***A***
2. ***A***^+^***AA***^+^ = ***A***^+^
3. (***AA***^+^)^*T*^ = ***AA***^+^
4. (***A***^+^***A***)^*T*^ = ***A***^+^***A***
5. *If **A** has full column rank, then **A***^+^ = (***A**^T^**A***)^−1^ ***A**^T^ (left inverse) and **A***^+^ ***A*** = ***I***.
6. *If **A** has full row rank, then **A***^+^ = ***A**^T^*(***AA**^T^*)^−1^ (*right inverse*) *and **AA***^+^ = ***I***.
7. *The MPP solves the “least squares” problem such that*
  $${∥Ax-b∥}_{2}\geq {∥Ax_{p}-b∥}_{2},\;\forall x\in R^{n},\;x_{p}=A^{+}b.$$

## Methods

### Relation between task and joint space quantities

This section focuses on the study of kinematic redundancy, which in turn will pave the way towards addressing the dynamic redundancy. Task space projection, a method that can reveal the properties of kinematic redundancy, has been thoroughly studied [19, 22] and we will briefly introduce the basic concept here. Establishing a link between task execution (e.g., movement of the hand) and muscle coordination is essential, as it is more convenient to interpret observations in task space rather than joint space. Furthermore, it is of great importance to transform the complex movement of the musculoskeletal system as a composition of multiple interleaved task goals, which may then be studied separately [24].

The task space position (***x***_*t*_) is given as a function of the generalized coordinates (***q***)

$$\begin{array}{c} x_{t}=g\left(q\right),\;x_{t}\in R^{d},\;q\in R^{n},\;d\leq n \end{array}$$ (8)

implying that the generalized coordinates fully describe the task. A task has an abstract meaning, it can be interpreted either as a position, an orientation or a spatial primitive, which is a combination of position and orientation. The first derivative of Eq (8) (the dot notation depicts a derivative with respect to time) is given by

$${\dot{x}}_{t}=J_{t}\left(q\right)\dot{q},\;J_{t}\left(q\right)=\left[\begin{array}{ccc} \frac{\partial g_{1}}{\partial q_{1}} & \cdots & \frac{\partial g_{1}}{\partial q_{n}}\\ \vdots & \ddots & \vdots \\ \frac{\partial g_{d}}{\partial q_{1}} & \cdots & \frac{\partial g_{d}}{\partial q_{n}} \end{array}\right]$$ (9)

where the task Jacobian matrix ($J_{t}\in R^{d\times n}$) defines a mapping from joint to task space ($R^{n}\to R^{d}$) and in most cases is a fat matrix. The inverse of Eq (9) must be augmented to account for the kinematic redundancy (Eq (7)) when mapping from task space ($R^{d}$—low-dimensional) to joint space ($R^{n}$—high-dimensional)

$$\begin{array}{c} \dot{q}={\dot{q}}_{∥}+{\dot{q}}_{⊥}=J_{t}^{+}{\dot{x}}_{t}+N_{F_{J_{t}}}{\dot{q}}_{0},\;N_{F_{J_{t}}}=\left(I-J_{t}^{+}J_{t}\right) \end{array}$$ (10)

where $N_{F_{J_{t}}}\in R^{n\times n}$ represents the right null space of ***J***_*t*_ (Definition 1) and ${\dot{q}}_{0}$ an arbitrarily selected vector in $R^{n}$. The derivative of Eqs (9) and (10) with respect to time are provided below

$$\begin{array}{c} {\ddot{x}}_{t}={\dot{J}}_{t}\dot{q}+J_{t}\ddot{q} \end{array}$$ (11)

$$\begin{array}{c} \ddot{q}={\dot{J}}_{t}^{+}{\dot{x}}_{t}+J_{t}^{+}{\ddot{x}}_{t}+{\dot{N}}_{F_{J_{t}}}{\dot{q}}_{0}+N_{F_{J_{t}}}{\ddot{q}}_{0} \end{array}$$ (12)

where ${\ddot{q}}_{0}$ is also an arbitrarily selected vector in $R^{n}$.

The task Jacobian defines a dual relation between motion and force quantities. The virtual work principle can be used to establish the link between task and joint space forces

$$\begin{array}{c} \begin{array}{cc} \tau^{T}\delta q & =f_{t}^{T}\delta x_{t}\\ \tau^{T}\delta q & =f_{t}^{T}J_{t}\delta q\\ \tau & =J_{t}^{T}f_{t} \end{array} \end{array}$$ (13)

where $J_{t}^{T}:R^{d}\to R^{n}$ defines a mapping from a low- to high-dimensional space. With the addition of the null space forces, the relationship between task forces and manipulator joint space generalized forces takes the following general form

$$\begin{array}{c} \tau =J_{t}^{T}f_{t}+\left(I-\underset{J_{t}^{+}J_{t}}{\underset{︸}{{\left(J_{t}^{+}J_{t}\right)}^{T}}}\right)\tau_{0}=J_{t}^{T}f_{t}+N_{F_{J_{t}}}\tau_{0} \end{array}$$ (14)

$$\begin{array}{c} f_{t}=J_{t}^{+T}\tau \end{array}$$ (15)

where $\tau_{0}\in R^{n}$ is an arbitrarily selected vector and Eq (15) the inverse mapping of Eq (14). Note that the null space complements the kinematic and dynamic relations in the sense that there are several combinations of joint space velocities, accelerations and generalized forces that bear no effect to the corresponding task space velocities, accelerations and forces. Furthermore, this redundancy is explored by the same null space term ($N_{F_{J_{t}}}$), both at a motion and force level by a virtue of the MPP properties (Definition 2).

**Remark 1**. *[Task motion—force duality]*

*The projection operators derived from* $J_{t}^{+}$ *and* $J_{t}^{T}$ *span the same subspaces*.

*Proof*. Appendix “Task motion—force duality”.

### Task space equations of motion

In the previous section (“Relation between task and joint space quantities”), the various relations between motion and force quantities of the kinetically redundant system were established. The current section continues with the derivation of the EoMs that relate the task acceleration to task space forces. Let the joint space EoMs have the following form

$$\begin{array}{c} M\left(q\right)\ddot{q}+f\left(q,\dot{q}\right)=\tau \end{array}$$ (16)

where $M\in R^{n\times n}$ denotes the symmetric, positive definite joint space inertia mass matrix, *n* the number of model DoFs and $q,\dot{q},\ddot{q}\in R^{n}$ the joint space generalized coordinates and their derivatives with respect to time. The term $f\in R^{n}$ represents the sum of all forces that act on the system (e.g., gravity, Coriolis, centrifugal, external, etc.), whereas $\tau \in R^{n}$ the vector of applied generalized forces that actuate the model.

We can project Eq (16) onto the task space by multiplying both sides from the left with ***J***_*t*_ ***M***^−1^ and using Eqs (11) and (14)

$$\begin{array}{c} J_{t}M^{-1}M\ddot{q}+J_{t}M^{-1}f=J_{t}M^{-1}\tau \\ {\ddot{x}}_{t}-{\dot{J}}_{t}\dot{q}+J_{t}M^{-1}f=J_{t}M^{-1}\left(J_{t}^{T}f_{t}+N_{F_{J_{t}}}\tau_{0}\right)\\ \Lambda_{t}\left({\overset{¨}{x}}_{t}+b_{t}\right)+{\bar{J}}_{t}^{T}f=f_{t}+{\bar{J}}_{t}^{T}N_{F_{J_{t}}}\tau_{0} \end{array}$$ (17)

where $\Lambda_{t}={\left(J_{t}M^{-1}J_{t}^{T}\right)}^{-1}\in R^{d\times d}$ denotes the task space inertia mass matrix, $b_{t}=-{\dot{J}}_{t}\dot{q}$ the task bias term and ${\bar{J}}_{t}^{T}=\Lambda_{t}J_{t}M^{-1}\in R^{d\times n}$ the generalized inverse transpose of ***J***_*t*_ that is used to project joint space quantities in the task space.

At this point it is worth commenting on the different types of null space operators. If the null space is derived using the MPP (as in Eq (14)), then the projection operator $N_{F_{J_{t}}}$ does not ensure fully decoupled control in both task and null space. It has been shown that from the many projection operators that map on the null space [25], there exists a unique generalized inverse (${\bar{J}}_{t}^{T}$) that ensures this decoupling [19], which is a useful property in the presence of multiple prioritized tasks [26]. More specifically, suppose that

$$\begin{array}{c} \tau =J_{t}^{T}f_{t}+\left(I-J_{t}^{T}{\bar{J}}_{t}^{T}\right)\tau_{0} \end{array}$$ (18)

then we seek to find the generalized inverse that ensures zero contribution of the null space forces to the acceleration of a task

$$\begin{array}{c} \Lambda_{t}\left({\overset{¨}{x}}_{t}+b_{t}\right)+{\bar{J}}_{t}^{T}f=f_{t}\\ J_{t}M^{-1}\left(I-J_{t}^{T}{\bar{J}}_{t}^{T}\right)\tau_{0}\overset{!}{=}0,\forall \tau_{0} \end{array}$$ (19)

where the last requirement ($\overset{!}{=}$) implies that

$$\begin{array}{c} {\bar{J}}_{t}^{T}=\Lambda_{t}J_{t}M^{-1}. \end{array}$$ (20)

**Remark 2**. *[Task space generalized inverse]*

*The null space projection operator* $N_{F_{J_{t}}}=I-J_{t}^{+}J_{t}$ *cannot decouple task and null space forces, owing to the uniqueness of the generalized inverse Jacobian that achieves this decoupling*.

*Proof*. [19].

### Relation between muscle and joint space quantities

Interestingly, we can follow a similar approach by establishing the corresponding relations between the muscle and joint space quantities in order to address the dynamic redundancy problem.

The musculotendon length is given as a function of the generalized coordinates (***q***)

$$\begin{array}{c} l_{m}=f\left(q\right),\;l_{m}\in R^{m},\;q\in R^{n},\;n<m \end{array}$$ (21)

implying that the generalized coordinates fully define the musculotendon lengths, assuming that the muscles are pretensioned. The derivative of Eq (21) with respect to time is given by

$${\dot{l}}_{m}=R(q)\dot{q},R(q)=\left[\begin{array}{ccc} \frac{\partial f_{1}}{\partial q_{1}} & \cdots & \frac{\partial f_{1}}{\partial q_{n}}\\ \vdots & \ddots & \vdots \\ \frac{\partial f_{m}}{\partial q_{1}} & \cdots & \frac{\partial f_{m}}{\partial q_{n}} \end{array}\right]$$ (22)

where the muscle moment arm ($R:R^{n}\to R^{m}$) is always a tall matrix. Due to the over-availability of muscles, Eq (22) can be reformulated to account for any change in ${\dot{l}}_{m}$ that does not contribute to a change in $\dot{q}$

$$\begin{array}{c} {\dot{l}}_{m}={\dot{l}}_{m}^{∥}+{\dot{l}}_{m}^{⊥}=R\dot{q}+N_{T_{R}}{\dot{l}}_{m0},\;N_{T_{R}}=\left(I-RR^{+}\right) \end{array}$$ (23)

$$\begin{array}{c} \dot{q}=R^{+}{\dot{l}}_{m} \end{array}$$ (24)

where $N_{T_{R}}\in R^{m\times m}$ represents the null space of ***R***, ${\dot{l}}_{m0}$ an arbitrarily selected vector in $R^{m}$ and Eq (24) the inverse mapping of Eq (23). The derivative of Eqs (23) and (24) with respect to time are given by

$$\begin{array}{c} {\ddot{l}}_{m}=\dot{R}\dot{q}+R\ddot{q}+{\dot{N}}_{T_{R}}{\dot{l}}_{m0}+N_{T_{R}}{\ddot{l}}_{m0} \end{array}$$ (25)

$$\begin{array}{c} \ddot{q}={\dot{R}}^{+}{\dot{l}}_{m}+R^{+}{\ddot{l}}_{m} \end{array}$$ (26)

with ${\ddot{l}}_{m0}$ being an arbitrarily selected vector in $R^{m}$. The null space contribution indicates that the CNS may regulate the lengthening/shortening of the muscles without altering the corresponding joint space quantities. This property can be used in coordinating different aspects of the tendon driven limbs or in the modeling of several pathological conditions of the muscle coordination process, such as slack muscle disorder.

From the virtual work principle (as in Eq (13)) we can establish a relationship between joint and muscle space forces

$$\begin{array}{c} \begin{array}{cc} \tau^{T}\delta q & =-f_{m}^{T}\delta l_{m}\\ \tau^{T}\delta q & =-f_{m}^{T}R\delta q\\ \tau & =-R^{T}f_{m} \end{array} \end{array}$$ (27)

where $R^{T}:R^{m}\to R^{n}$. The negative sign convention is introduced, admitting that a muscle induces positive work during shortening (${\dot{l}}_{m}≺0$). The muscle forces ***f***_*m*_ can be written as the sum of the two mutually orthogonal vectors $f_{m}^{∥}\in C\left(R^{+T}\right)$ and $f_{m}^{⊥}\in N\left(R^{+}\right)$ (Eq (7)). In contrast to Eq (27), the direct mapping does not require any reformulation, but the inverse requires reformulation in order to account for the redundancy effect

$$\begin{array}{c} f_{m}=-R^{+T}\tau +\left(I-\underset{RR^{+}}{\underset{︸}{{\left(RR^{+}\right)}^{T}}}\right)f_{m0}=\underset{f_{m}^{∥}}{\underset{︸}{-R^{+T}\tau }}+\underset{f_{m}^{⊥}}{\underset{︸}{N_{T_{R}}f_{m0}}} \end{array}$$ (28)

where $N_{T_{R}}$ is the same matrix defined in Eq (23), revealing the dual relationship between the subspaces of ***R*** and ***R***^+*T*^ (Remark 3). Note, however, that this definition spans the entire $R^{m}$, whereas in reality muscle forces are strictly positive (contraction) and bounded (limited force), which makes the above definition physiologically ill conditioned. In section “Exploitation of kinematic and dynamic redundancy”, we will provide an effective solution to this problem. If a solution satisfying the required torque exists, then there are infinitely many combinations of muscle forces ($N_{T_{R}}f_{m0}$) that do not alter the overall required torque. On a physiological level, this strategy is explored by the CNS in order to regulate joint stiffness through muscle co-contraction [7].

**Remark 3**. *[Moment arm motion—force duality]*

*The projection operators of **R** defines the same subspaces as the projection operators of **R***^+*T*^.

*Proof*. Similar to Appendix “Task motion—force duality”.

To summarize, it is of particular interest, whether pathological conditions can be encoded in the choice of ${\dot{l}}_{m0}$, ${\ddot{l}}_{m0}$ and ***f***_*m*0_. Examples include the investigation of the cause-effect of slack muscle tone (e.g., hypotonia or hypertonia) or the increase in co-contraction causing rigidity. The incorporation of the null space complements the original equations and unveils the structure of dynamic redundancy. Additional steps have to be performed to ensure that Eq (28) provides physiologically correct interpretations.

### Muscle space equations of motion

In the previous section (“Relation between muscle and joint space quantities”), the various relations between motion and force quantities of the dynamically redundant system were established. In this section we will derive the EoMs that relate the muscle lengthening/shortening accelerations to muscle space forces.

As was previously shown (Eq (28)), the muscle forces ***f***_*m*_ can be expressed as the sum of two mutually orthogonal subspaces that span $R^{m}$ ($f_{m}=f_{m}^{∥}+f_{m}^{⊥}$). We can project Eq (16) onto the muscle space by multiplying both sides from the left with −***RM***^−1^, also taking Eqs (25) and (27) into account

$$\begin{array}{c} -RM^{-1}M\ddot{q}-RM^{-1}f=-RM^{-1}\tau \\ -({\ddot{l}}_{m}-\dot{R}\dot{q}-{\dot{N}}_{T_{R}}l_{m0}-N_{T_{R}}{\overset{¨}{l}}_{m0})-RM^{-1}f=RM^{-1}R^{T}f_{m}\\ -\Lambda_{m}({\ddot{l}}_{m}+b_{m})-R^{+T}f=f_{m}^{\|}+N_{T_{R}}f_{m0} \end{array}$$ (29)

where $\Lambda_{m}={\left(RM^{-1}R^{T}\right)}^{+}\in R^{m\times m}$ represents the muscle space inertia mass matrix, $b_{m}=-\dot{R}\dot{q}-{\dot{N}}_{T_{R}}{\dot{l}}_{m0}$ the muscle bias term and $\Lambda_{m}RM^{-1}=R^{+T}\in R^{m\times n}$ is used to project joint space quantities onto the muscle space. Note that the null space term $N_{T_{R}}{\ddot{l}}_{m0}$ cancel out when multiplied by $\Lambda_{m}$ (Proposition 1). When Eq (29) is solved in an Inverse Dynamics (ID) manner, the quantities of interest are the muscle forces ***f***_*m*_, while ${\dot{l}}_{m0}$, ***f***_*m*0_ are free variables which are selected to satisfy additional modeling criteria.

**Proposition 1**. *[Muscle space inertia mass matrix]*

*Given that **T**_**R**_* = ***RR***^+^ *and* $\Lambda_{m}={\left(RM^{-1}R^{T}\right)}^{+}$ *the following assertions hold*:

1. **Λ**_*m*_ *is symmetric* ($\Lambda_{m}=\Lambda_{m}^{T}$) *iff **M** is symmetric*.
2. **Λ**_*m*_ *remains invariant under the projection operator **T**_**R**_*.
3. $\Lambda_{m}\Lambda_{m}^{+}=T_{R}$.
4. $\Lambda_{m}N_{T_{R}}=0$.

*Proof*. Appendix “Muscle space inertia mass matrix”.

The introduction of the null space term ($N_{T_{R}}f_{m0}$) complements the relationship between muscle and joint space forces (Eq (28)) and the muscle space EoMs (Eq (29)). Without this term, the relations are ill conditioned due to physiological restrictions of the muscles (**0** ≼ ***f***_*m*_ ≼ ***f***_*max*_). Although a particular solution ***f***_*m*_ or $f_{m}^{∥}$ for some arbitrary τ or ${\ddot{l}}_{m}$, respectively does not necessarily satisfy these restrictions, a suitable ***f***_*m*0_ can be selected. Furthermore, several aspects and patterns of muscle co-contractions can be modeled by appropriately selecting ***f***_*m*0_.

### Exploitation of kinematic and dynamic redundancy

In the previous section, we derived the EoMs in muscle space. Furthermore, we decomposed the muscle forces (***f***_*m*_) into two mutually orthogonal subspaces that span the entire muscle space ($R^{m}$). Here we will define an effective approach for taking advantage of the null space contribution in order to satisfy the physiological restrictions of the muscles.

Muscle redundancy manifests itself during ID, whereas the null space term does not contribute to the movement in a Forward Dynamics (FD) setting, because $R^{T}N_{T_{R}}f_{m0}=0$. For a given action a particular solution of muscle forces ($f_{m}^{∥}$) can be found by initially ignoring the null space contribution ($f_{m}^{⊥}$), which is not guaranteed to be physiologically correct (namely $\exists i:f_{m}^{i}>f_{max}^{i}\;\text{or}\;f_{m}^{i}<0$). However, the null space can provide a suitable correction in order to satisfy the physiological constraints. A solution of muscle forces $f_{m}^{∥}$ in the task, joint or muscle space respectively can be obtained as follows (note that the MPP solves the least squares problem Definition 2)

$$\begin{array}{c} f_{m}^{\|}=-\Theta^{T}\left(\Lambda_{t}{\ddot{x}}_{t}+b_{t}\right)+{\bar{J}}_{t}^{T}f),\Theta =J_{t}R^{+}\in {ℜ}^{dxn}\\ f_{m}^{\|}=-R^{+T}(M\ddot{q}+f)\\ f_{m}^{\|}=-\Lambda_{m}\left({\ddot{l}}_{m}+b_{m}\right)-R^{+T}f. \end{array}$$ (30)

It is well-known that muscle dynamics are described by a complex nonlinear system [27–29] that depends on a multitude of factors, such as the strength of the muscles, the muscle activation dynamics, the pennation angle, the elastic properties of the muscle fibers and tendon, the force-length and the force-velocity characteristics, etc. Consequently, these factors will also affect the null space solution component ***f***_*m*0_. Assuming the simplest possible muscle model

$$\begin{array}{c} f_{m}=f_{max}\circ a_{m},\;0≼a_{m}≼1 \end{array}$$ (31)

where $a_{m}\in R^{m}$ represents a vector of muscle activations, $f_{max}\in R^{m}$ a vector specifying the maximum muscle forces and ∘ the Hadamard (elementwise) product. We can impose bounds on the possible solutions of ***f***_*m*0_ in the form of linear inequalities, by noting that Eqs (28) and (31) must be equal

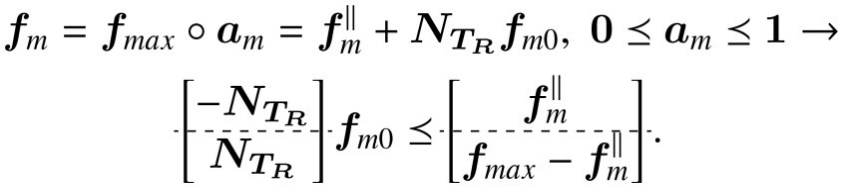
(32)

Given an appropriately selected cost function, a unique solution of the linear inequalities can be obtained by solving the constrained optimization problem. A generalization for a nonlinear muscle model [27] is presented below

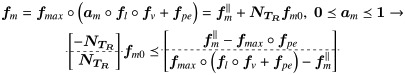
(33)
where $f_{l},\;f_{v},\;f_{pe}\in R^{m}$ are the force-length, force-velocity and passive muscle forces.

It is of great interest to understand the structural dependencies of these inequalities in order to outline the feasible muscle force space. For a muscle model M and an arbitrary action A, the problem is expressed in its most general form (generalization of Eq (33)):

$$\begin{array}{c} Z\left(N_{T_{R}}\right)f_{m0}≼\beta \left(M,\;A\right) \end{array}$$ (34)

where the solutions (***f***_*m*0_) lie in a convex subspace of $R^{r}$ (*r* ≤ *m*) and the feasible space is bounded (Proposition 2). Note that the action (A) implicitly encodes the kinematic redundancy (Eq (30)). Finally, the feasible muscle force set is computed as the sum of the particular solution and the null space forces that satisfy the inequalities

$$\begin{array}{c} f_{m}^{⊕}=\{f_{m}^{∥}+N_{T_{R}}f_{m0}^{i},\;\forall i\}. \end{array}$$ (35)

**Proposition 2**. *[Feasible null space forces]*

*The following properties hold true*:

1. Eq (34) *defines a convex set* C.
2. Eq (34) *is bounded if the inequality is feasible and if **Z** is full column rank*.

*Proof*. Appendix “Feasible muscle force space”.

Traditional solutions of the muscle redundancy problem [1] (e.g., effort minimization) ignore the possibilities of the system and prove to be inadequate in quantifying the decisive factors, whereas Eqs (34) and (35) have an elegant form that outlines the entire set of possible solutions. More importantly, each solution can be characterized (e.g., minimum effort, high stiffness, rigidity, etc.), enabling classification of the different manifolds (strategies) available to the CNS.

**Fig 2:**
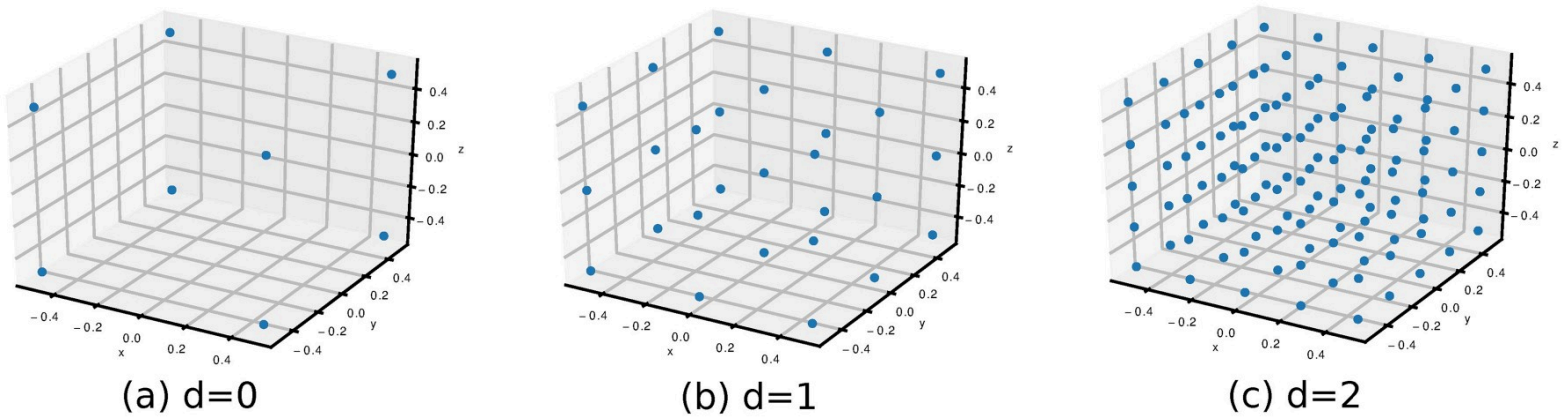
Sampling of bounded inequality. The inequality Eq (36) is sampled using Algorithm 1 for different levels of iteration depth (*d*).

In order to compute the feasible muscle force set $f_{m}^{⊕}$, the physiological null space muscle forces ***f***_*m*0_ must be obtained by sampling the space defined by a set of linear inequalities (Eq (34)). The linear inequalities define a polytope (a convex polyhedron) as an intersection of a finite number of half-spaces (hyperplane- or H-representation). According to Proposition 2, this polytope has a finite number of extreme points (vertex- or V-representation), which we use to sample this polytope sufficiently. The conversion from H-representation to V-representation is called vertex enumeration and can be achieved by using either a deterministic [30] or randomized [31] approach. From an arrangement of *n* hyperplanes in $R^{d}$, *v* vertices are determined in *O*(*n*^2^ *dv*) time. In our problem *n* = 2 *m*, *d* ≤ *m* (*m* being number of muscles), thus the time complexity is *O*(*vm*^3^). In this work, we used the lrs library [30], which provides a self-contained ANSI C implementation of the reverse search algorithm for vertex enumeration. After obtaining the extreme points of the polytope, additional solutions are generated by interpolating (ψ=λx+(1-λ)y) between vertices. This process generates samples spanning the entire polytope due to its convexity, as proved earlier. An example of Algorithm 1 is presented (Fig 2) for the following system

$$Ax\leq b,A=\left[\begin{array}{ccc} 0 & 0 & -1\\ 0 & -1 & 0\\ 1 & 0 & 0\\ -1 & 0 & 0\\ 0 & 1 & 0\\ 0 & 0 & 1 \end{array}\right],b=\left[\begin{array}{c} 0.5\\ 0.5\\ 0.5\\ 0.5\\ 0.5\\ 0.5 \end{array}\right].$$ (36)

**Algorithm 1** Iterative sampling of a convex bounded polyhedron.

**Input**: H-representation ***Ax*** ≤ ***b***, *d*: iteration depth

**Output**: A representative set S={x:Ax≤b}

1: Find the V-representation V using lrs (or some other method)

2: **for** *i* = 0 **to** *d* **do**

3:  T=V

4:  **for all** distinct {x,y}∈T **do**

5:   Add ψ=λx+(1-λ)y to V {λ = 0.5}

6:  **end for**

7: **end for**

8: **return** V

## Results

We proceed by demonstrating working examples of the developed models. Section “Muscle space projection and reflexes” presents the utilization of muscle space projection for solving the posture control problem, where the muscle length and derivatives are the regulating quantities. This experiment highlights the importance of coordinate projection, which essentially transforms the EoMs onto some space (e.g., task or muscle) to enable direct control of the quantity of interest. Subsequently, section “Characterization of the feasible muscle force space” continues by evaluating the feasible muscle forces in the context of an action and identifying force variabilities and muscle group correlations. We highlight the main contribution of this work, which is the calculation of the feasible muscle forces that satisfy both the task and the physiological constraints of the muscles. The two experiments follow a Mixed Dynamics (MD) scheme, where the FD method is used for simulating the model combined with an ID model-based controller (muscle and task space, respectively), representing the underlying system. These results are included as a mere demonstration of the possibilities that arise under our framework using simple examples. Last but not least, a joint reaction analysis is performed on a gait movement, appropriately accounting for the null space muscle forces that do not alter the movement, demonstrating the importance of estimating the null space contribution and its impact on the joint reaction loads.

**Fig 3:**
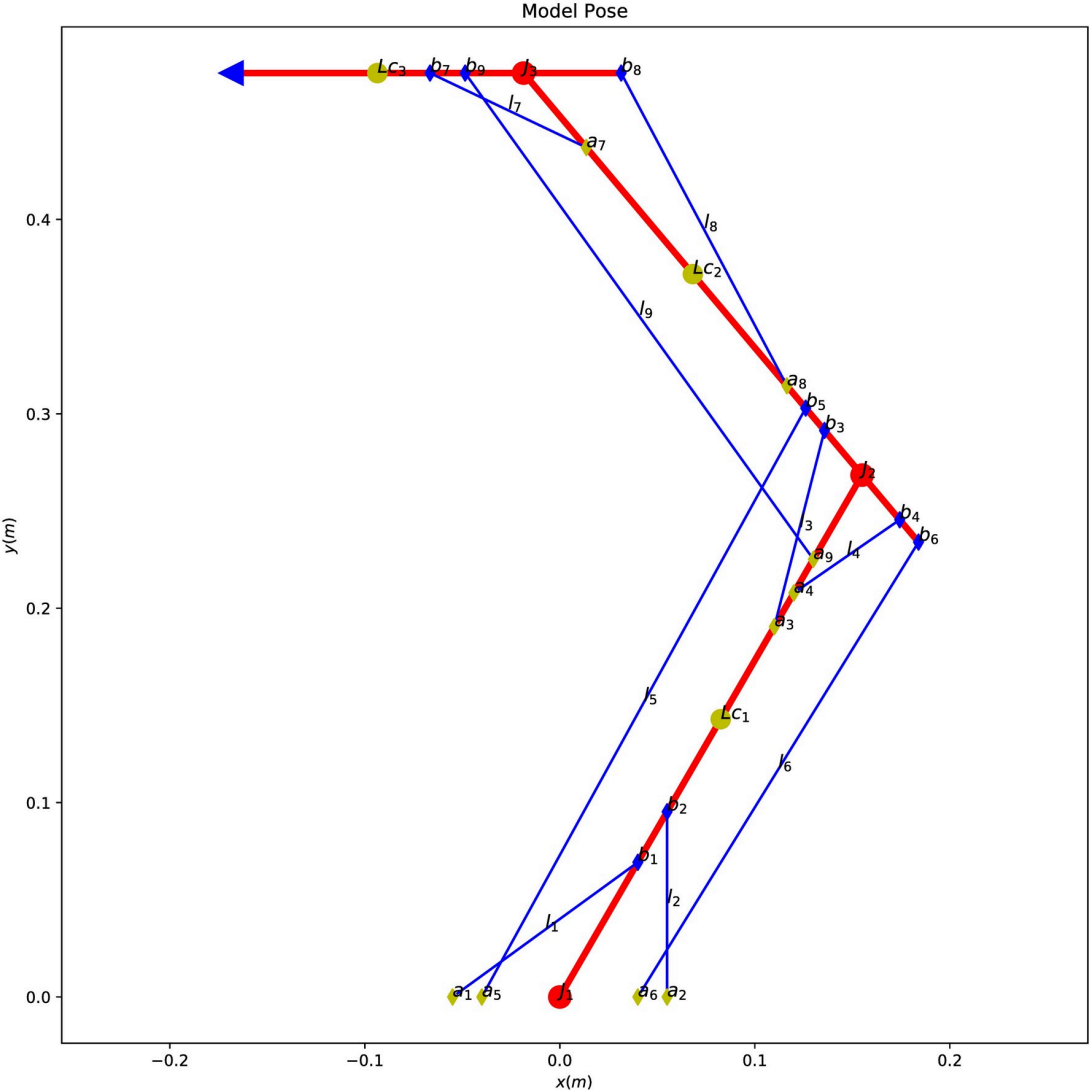
Diagram of the simplified arm model. The model has three DoFs and nine muscles, some of them being bi-articular. The muscle origins are labeled as *a*_*i*_ and the muscle insertions as *b*_*i*_. *l*_*i*_ stands for muscle length, *Lc*_*i*_ for center of mass and *J*_*i*_ for joint center.

A simplified arm model has been developed in order to demonstrate the utilization of the proposed methods. It has three DoFs and nine muscles (Fig 3), appropriately constructed to demonstrate both kinematic and dynamic redundancy (e.g., *d* < *n* < *m*). The EoMs and the geometric parameters of the model are derived analytically in order to evaluate higher order derivatives of moment arm and task Jacobian matrices (S1 File). The model is able to capture the redundancy effects conveying the main contribution of this work and is also an excellent tool for introducing these methods. An anatomically realistic full body gait model [32] is used for the joint reaction analysis (OpenSim [33]), illustrating that the presented methods are universal and can be applied in more practical and realistic scenarios. The model, which has ten DoFs and eighteen muscles (Millard muscle model [29]), features lower extremity joint definitions, low back joint and a planar knee model.

### Muscle space projection and reflexes

Muscle space projection exhibits several advantages in problems related to the segmental level coordination, mainly because of the different muscle related variables (e.g., length, velocity, muscle stiffness) that are directly/indirectly controlled by the CNS [11, 12]. For example, the proprioceptive receptors, located in the spinal cord, constantly measure the evolution of these variables and issue corrective actions [34]. More specifically, the muscle spindle organs [35] measure changes in the muscle length and the Golgi tendon organs [36] measure the force exerted by their respective muscles. This indicates that the aforementioned variables are used in an internal feedback regulation process [3].

We continue with the design of a posture controller that coarsely encapsulates the characteristics of the internal regulation process performed in the spinal cord and the evaluation of system’s response to external disturbances. As the muscle length and its derivative are the regulating variables, muscle space projection is a natural representation in this setting. The following control scheme is adopted for this experiment

$$\begin{array}{c} {\ddot{l}}_{m}\left(t\right)=k_{p}\left(l_{m}^{d}-l_{m}\left(t-\tau_{so}\right)\right)-k_{d}{\dot{l}}_{m}\left(t-\tau_{so}\right) \end{array}$$ (37)

where ${\ddot{l}}_{m}\left(t\right)$ represents the muscle length acceleration goals, $l_{m}^{d}=l_{m}\left(t=0\right)$ the desired muscle length positions, ***l***_*m*_(*t* − *τ*_*so*_) and ${\dot{l}}_{m}\left(t-\tau_{so}\right)$ the perceived, delayed (*τ*_*so*_ = 20 ms originating from muscle spindle organs [3]) muscle length positions and velocities and *k*_*p*_, *k*_*d*_ the reflex gains. Note that this control law restores the system to its original posture ***l***_*m*_(*t* = 0). A force disturbance impulse in an arbitrary direction is applied on the end effector body. The impulse is modeled by a Gaussian function

$$\begin{array}{c} f\left(t\right)=ae^{\frac{-(t-t_{0})}{2\sigma^{2}}} \end{array}$$ (38)

where *a* controls the magnitude, *t*_0_ the application time and *σ* the smoothness of the impulse. For the following experiments we will use *a* = 15, *t*_0_ = 0.1, *σ* = 0.01 and the perturbation will act in the −***x*** direction. The muscle space EoMs Eq (29) can serve as an ID model-based controller for calculating the required muscle forces ***f***_*m*_ (Algorithm 2), which may then be fed to the FD module. The ${\dot{l}}_{m0}$ in Algorithm 2 can be arbitrarily selected by the modeler to encode various strategies available by the CNS. In our experiments, ${\dot{l}}_{m0}$ is set zero to encode an unbiased strategy, for the sake of simplicity.

**Algorithm 2** Muscle space force computation for a given goal using muscle space projection.

**Input**: muscle length accelerations ${\ddot{l}}_{m}$, null space muscle length velocities ${\dot{l}}_{m0}$

**Output**: muscle forces ***f***_*m*_

1: Solve Eq (29) for $f_{m}^{∥}$ by initially ignoring the null space term $N_{T_{R}}f_{m0}$

2: Minimize ||***f***_*m*0_||^2^ for ***f***_*m*0_ subject to Eq (34)

3: Obtain the required muscle forces as $f_{m}=f_{m}^{∥}+N_{T_{R}}f_{m0}$

4: **return *f***_*m*_

**Fig 4:**
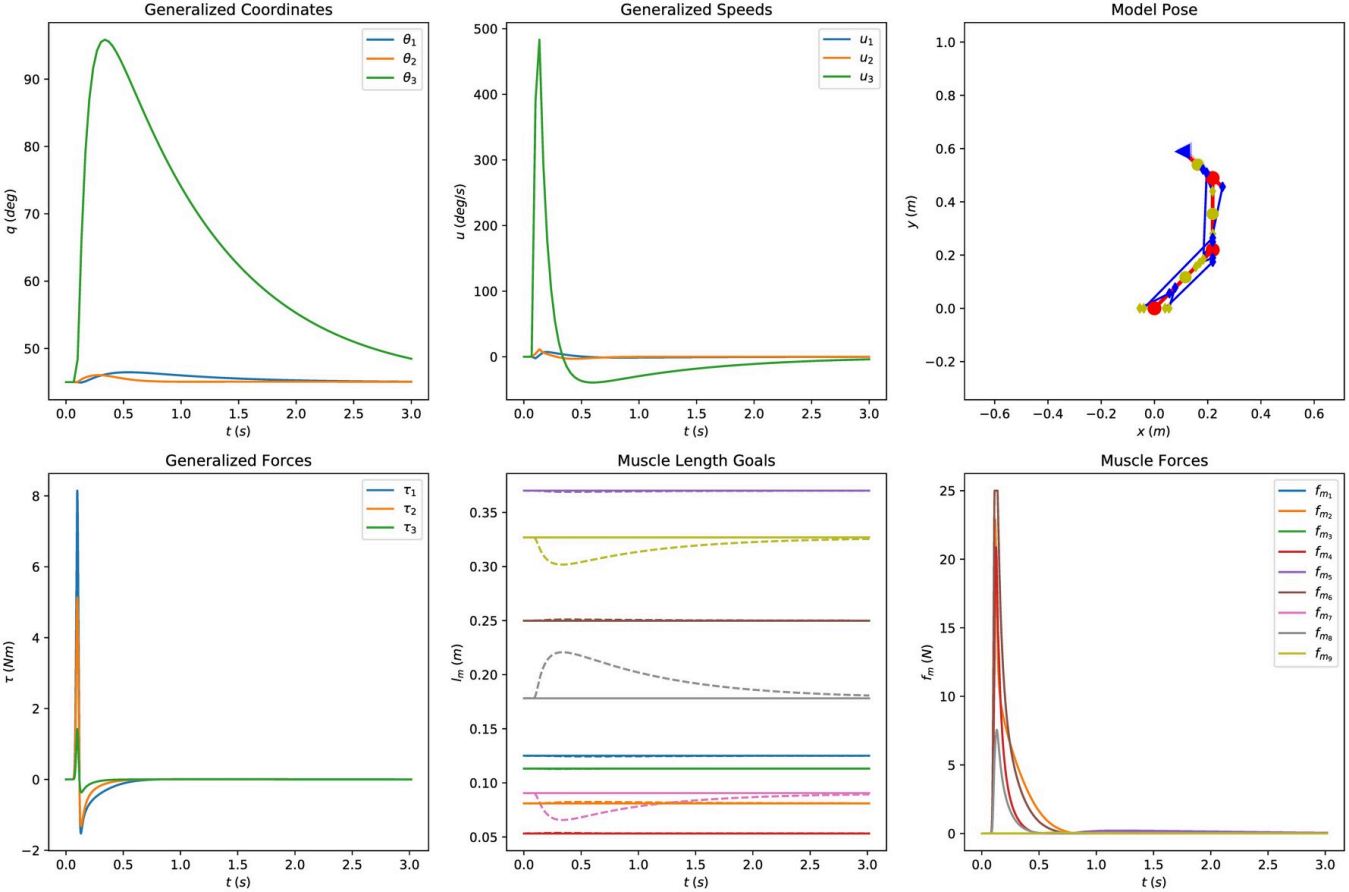
Posture analysis of the system in muscle space, including a “descending” command from the CNS. The first row of figures presents the simulated joint space coordinates and speeds. The second row depicts the evolution of the muscle space quantities of the controller. The model is perturbed and the response of the system is observed. The system absorbs the impact and returns to its initial configuration. The controller gains are *k*_*p*_ = 10, *k*_*d*_ = 10 and the loop delay *τ*_*so*_ = 20 ms.

**Fig 5:**
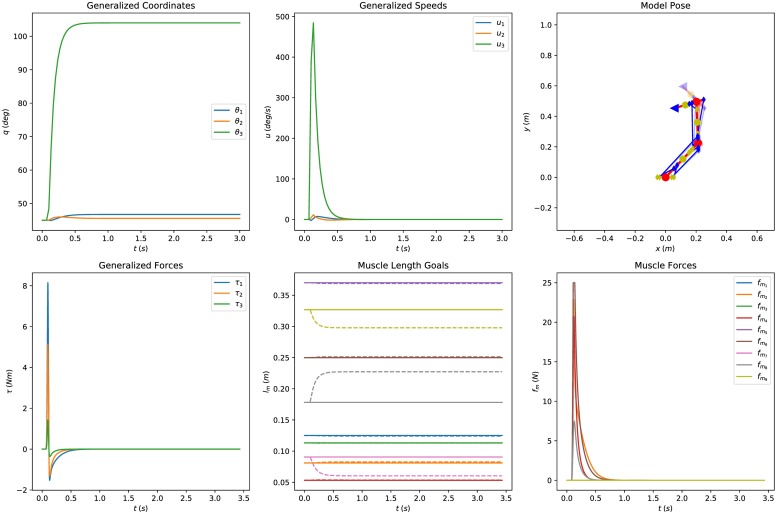
Posture analysis of the system in muscle space without “descending” command from the CNS. The first row of figures presents the simulated joint space coordinates and speeds. The second row depicts the evolution of the muscle space quantities of the controller. After the perturbation the system settles to a new, stable equilibrium point. The controller gains are *k*_*p*_ = 0, *k*_*d*_ = 10 and the loop delay *τ*_*so*_ = 20 ms.

Fig 4 depicts collectively the simulation results when the system is perturbed and the two reflex loops are active (*k*_*p*_ = 10, *k*_*d*_ = 10 and *τ*_*so*_ = 20 ms). The impact is absorbed and the system returns to its initial configurations (stable response). Conversely, in Fig 5 the “descending” command is disabled (*k*_*p*_ = 0), leaving the response of the reflex loop alone, in which the system reaches a new, stable equilibrium. The muscle space projection suits this kind of studies well as it offers a direct interface with segmental level models. On the contrary, additional transformation of the control variables is required if the problem were expressed in joint space. Different combinations of reflex loop gains, delays and external factors can be evaluated quantitatively, demonstrating the usefulness of this projection.

### Characterization of the feasible muscle force space

In this experiment, the feasible muscle forces that satisfy the task and the physiological muscle constraints are computed. The analysis is restricted on a particular time instance, where we identify the muscle force bounds and correlations between different muscle pairs. The movement of the hand is planned in task space using Eqs (18) and (19) as an ID model-based controller in combination with a Proportional Derivative (PD) tracking scheme

$$\begin{array}{c} {\ddot{x}}_{t}={\ddot{x}}_{d}+k_{p}\left(x_{d}-x_{t}\right)+k_{d}\left({\dot{x}}_{d}-x_{t}\right) \end{array}$$ (39)

where ***x***_*d*_, ${\dot{x}}_{d}$ and ${\ddot{x}}_{d}$ denote the desired position, velocity and acceleration of the end effector and *k*_*p*_ = 50, *k*_*d*_ = 5 the tracking gains. The planning problem can be encoded naturally in task space as compared to joint and muscle space controllers. Note that this analysis is independent of the underlying ID representation (Eq (30)). Furthermore, a linear muscle model (Eq (32)) is assumed.

The desired task goal is derived from a smooth sigmoid function that produces bell-shaped velocity profiles in any direction around the initial position of the end effector

$$\begin{array}{c} x_{d}(t)={\left[x_{t}(0)+\frac{a}{2}\left(\text{tanh}\left(b\left(t-t_{0}\right)\right)+1\right),y_{t}(0)\right]}^{T},{\dot{x}}_{d}(t)=\frac{dx_{d}(t)}{dt},{\ddot{x}}_{d}(t)=\frac{d{\dot{x}}_{d}(t)}{dt}\\ x_{d}^{\prime }=H_{z}(\gamma )x_{d},{\dot{x}}_{d}^{\prime }=H_{z}(\gamma ){\dot{x}}_{d},{\overset{¨}{x}}_{d}^{\prime }=H_{z}(\gamma ){\overset{¨}{x}}_{d} \end{array}$$ (40)

where *x*_*t*_, *y*_*t*_ represent the 2 *D* components of ***x***_*t*_, *a* = 0.3, *b* = 4 and *t*_0_ = 1. Different directions of movement are achieved by transforming the goals with ***H***_*z*_(*γ*), which defines a rotation around the ***z***-axis of an angle *γ*. Eqs (34) and (35) enable the exploration of the feasible muscle forces. In order to compute the feasible muscle force set $f_{m}^{⊕}$ (Eq (35)), Eq (34) must be sampled for ***f***_*m*0_. In this experiment, we compute the vertices of the polytope using Algorithm 1 with *d* = 0. The process is summarized in the following algorithm (Algorithm 3).

**Algorithm 3** Calculation of the feasible muscle forces as affected by the moment arm null space, muscle model and the action.

**Input**: action τ, muscle model M, null space $N_{T_{R}}$

**Output**: feasible muscle forces $f_{m}^{⊕}$

1: Find the particular muscle force solution $f_{m}^{∥}$ (Eq (30))

2: Sample Eq (34) for ***f***_*m*0_ (Algorithm 1)

3: Calculate the feasible muscle force set from Eq (35)

4: **return** $f_{m}^{⊕}$

**Fig 6:**
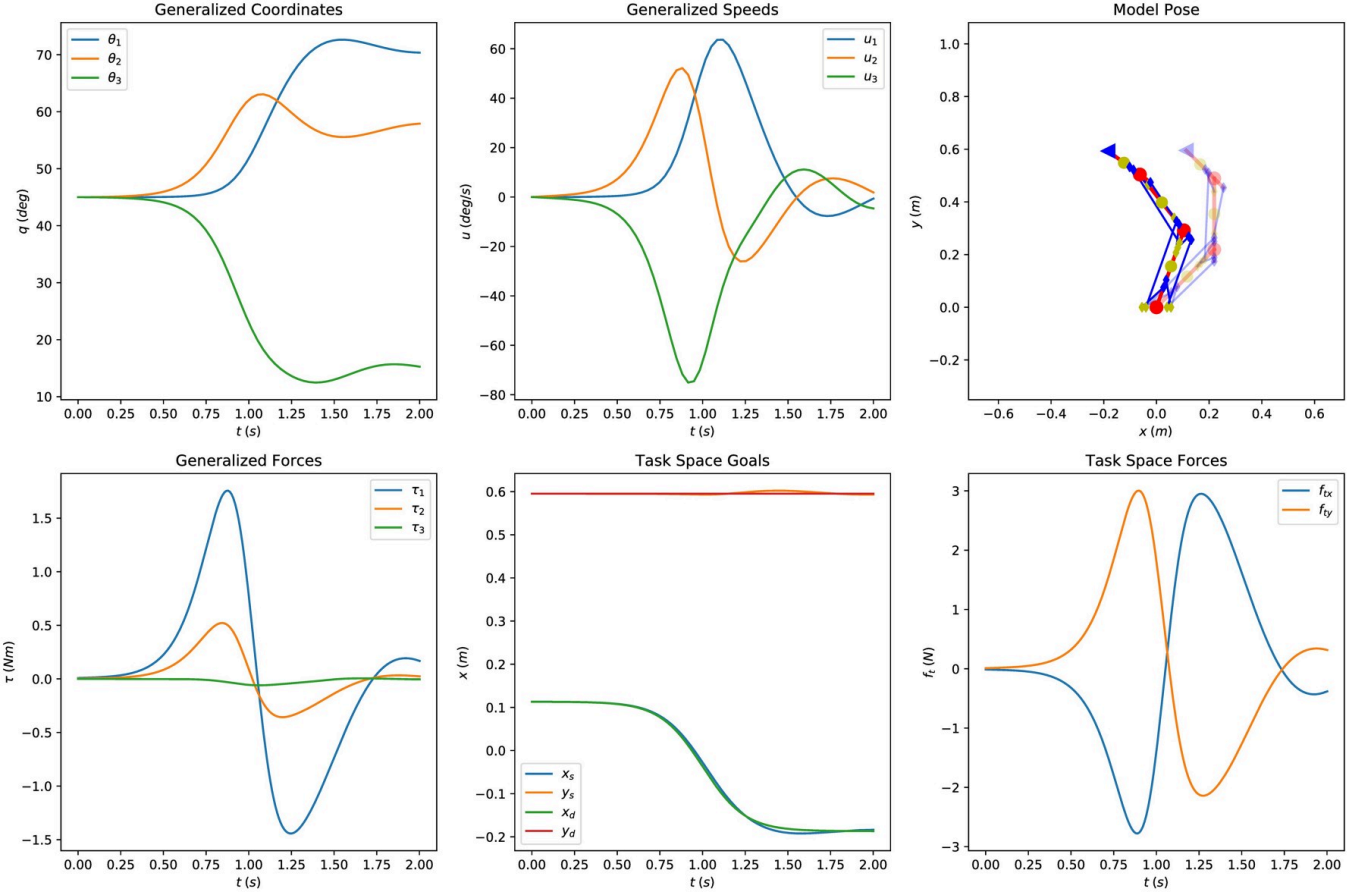
Collective simulation results for the simplified hand movement. The evolution of the general coordinates and speeds as well as the simulated movement are presented in the figures of the first row. The second row shows the generalized forces, the simulated and desired task goals and the task space forces computed by the controller. The parameters used in this experiment are *k*_*p*_ = 50, *k*_*d*_ = 5, *a* = 0.3, *b* = 4, *t*_0_ = 1 and *γ* = *π*.

**Fig 7:**
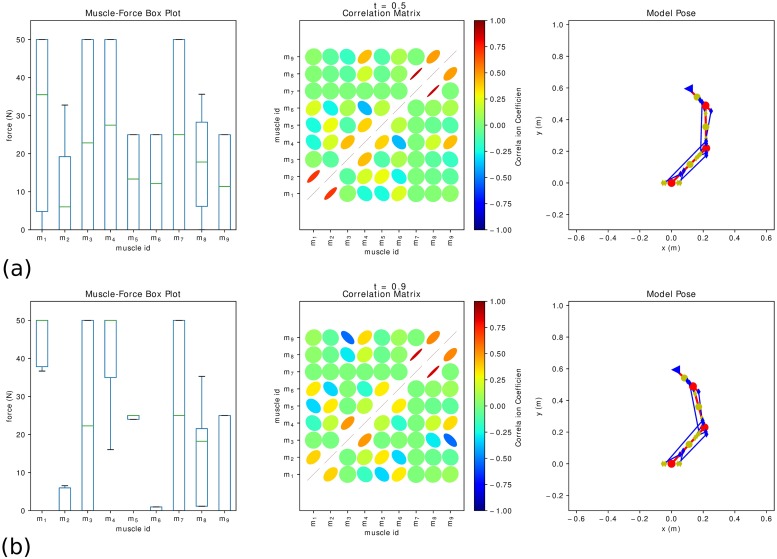
The evolution of the feasible force set. Two instances of the simulated movement along the −***x*** direction are shown to demonstrate the evolution of the feasible muscle space. The box plot depicts the force variability of each muscle as contributed to the redundant nature of the system. The correlation matrix shows the positive or negative correlation between the different muscle pairs.

Fig 6 presents the simulation results for the simplified hand movement (movement along the −***x*** direction, *γ* = *π*) collectively. We can inspect the evolution of the generalized coordinates and speeds as well as simulated/desired task goals and the corresponding task forces computed by the controller. Two instances of the simulation are isolated in Fig 7, where the feasible muscle forces that satisfy the task are further analyzed. The resulting feasible spaces are high-dimensional and various useful conclusions can be drawn. The feasible muscle force space changes as the movement progresses, since it depends on the action, the muscle model and the muscle routing properties.

The box plot shows the force variability of each muscle, which is attributed to the redundant nature of the system. From a control point of view, the variability of each muscle seems to be inversely proportional to the importance of its contribution to the current movement, following the Uncontrolled Manifold Hypothesis (UMH) [9]. Fig 7 presents two instances of the simulated movement, one at the onset of the movement (0.5 *s*) and one at the peak of the required effort (0.9 *s*), permitting a qualitative comparison the feasible muscle forces. As the force requirements are small in the beginning of the movement (Fig 7a), the feasible muscle forces span the entire space. On the contrary (Fig 7b), as the demand for movement increases the agonists retain higher force profiles, while the antagonists’ contribution is attenuated. The provided correlation matrix between different muscle groups reveals agonist-antagonist relations, which vary as the movement progresses. It can be concluded that there is a significant systematic correlation between different muscle pairs that emerges from the muscle routing properties and the functional constraints of the task.

### The effect of null space on the joint reaction loads

The importance of evaluating the feasible muscle forces is demonstrated in the context of joint reaction analysis. An accurate estimation of the muscle forces is essential for the assessment of joint reaction loads. Consequently, the null space contributions can significantly alter the reaction forces without affecting the movement.

A benchmark gait movement, available from the OpenSim dataset [33] was used for this analysis. In a typical experimental setup the motion and externally applied forces are recorded. Given these recordings, Inverse Kinematics (IK) and ID are performed in order to assess the model kinematics and kinetics required to track the experimental measurements [37]. Instead of estimating the muscle forces using Static Optimization (SO) or some other method [1, 38], we can recall Eq (28) and solve for ***f***_*m*_ accounting for the null space muscle forces, which are present only when projecting from low- to high-dimensional space. Different realizations of muscle forces were calculated by appropriately sampling the feasible muscle force space (Eqs (34) and (35)). Finally, multiple joint reaction analyses were performed to evaluate the effect of the feasible muscle forces on the reaction loads.

**Fig 8:**
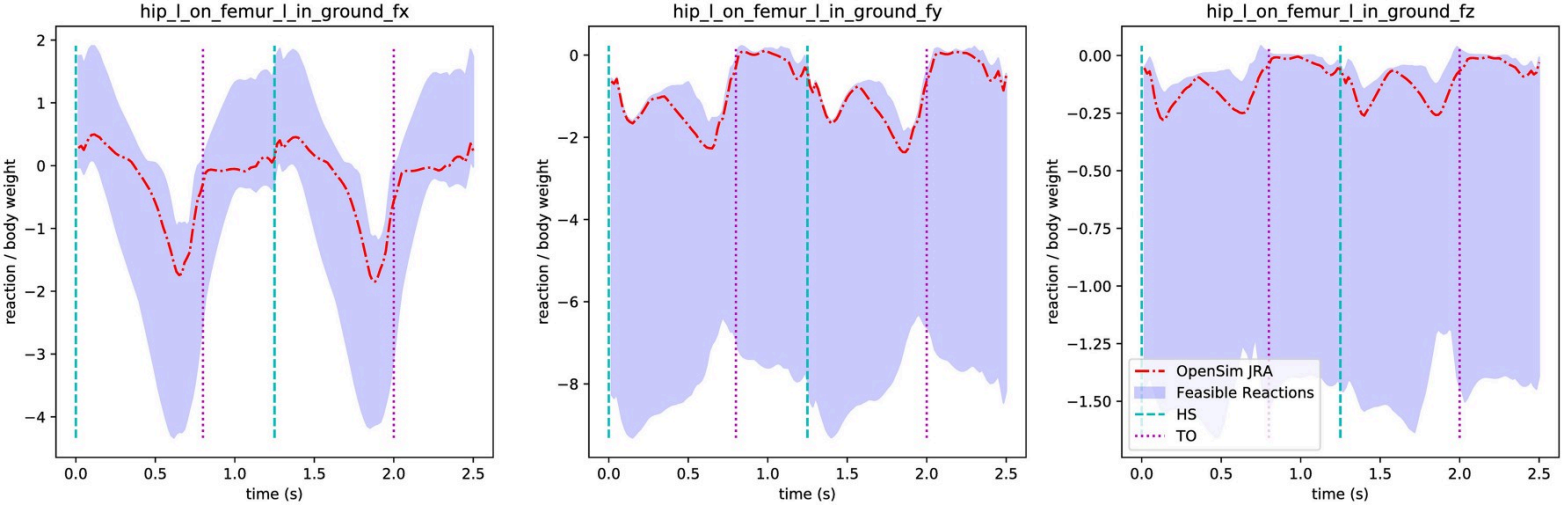
The effect of the null space muscle forces on the joint reaction loads. The normalized (with respect to body weight) reaction loads on the hip joint during walking are reported along with the heel strike and toe-off events. The shaded area is the reaction force range as attributed to the null space solutions of muscle forces. The red dotted line are the results obtained from OpenSim. The large range in the reaction loads confirms that misinterpretation of the results is possible if the null space solutions are ignored.

Fig 8 presents the normalized (with respect to body weight) reaction forces on the hip joint during walking with the heel strike and toe-off events annotated accordingly. We observe that the results obtained from OpenSim joint reaction analysis predict low reaction load levels, since the minimum muscle effort criterion is used to compute the muscle forces, ignoring muscle co-contraction. On the contrary, it is possible to calculate the feasible reactions without making any prior assumption, which can limit the scope and extent of the analysis. Last and perhaps most importantly, the large range of possible values confirms that misinterpretation of the results is possible if the null space solutions are ignored.

## Discussion

In this work, we approached the musculoskeletal redundancy problem by extending the kinematic and dynamic relationships using the notion of null space. This allowed us to study the problem more systematically and to avoid introducing unnecessary assumptions (e.g., muscle optimization). To achieve this level of modeling three operational spaces (task, joint and muscle space) were introduced, whose main purpose is to allow a distinction between the kinematic and dynamic redundancy effects. More specifically, the kinematically redundant DoFs in the context of a task were identified by considering the task and joint space relationships. Likewise, the relations between joint and muscle or task and muscle spaces were used to infer the level of muscle co-contraction, which result in the same movement behavior. From a theoretical perspective, the main contribution of this work is the modeling of musculoskeletal redundancy using the notion of null space and the calculation the feasible solution space, enabling various useful post-analyses (e.g., feasibility studies, joint reaction analysis, task/joint stiffness evaluation, etc.). The adoption of this framework can potentially enable the modeling and evaluation of hypotheses related to normal and pathological conditions presented in the coordination process, which can manifest in the null solution space (e.g., slack muscle disorder, increase in joint stiffness and rigidity).

From a control point of view, the purpose of projecting the EoMs onto some space is to enable the direct control of some quantity of interest. In this work, we used the various representations of the EoMs as ID model-based controllers in a MD scheme in order to perform FD simulations by controlling the task positions or muscle lengths. When dealing with models that possess many DoFs, their posture is usually controlled by several simultaneous tasks [23, 26]. Task space representation is preferred, since the planning is encoded more naturally compared to joint space (e.g., animation of characters). We introduced the muscle space projection where the controlled quantities are muscle lengths and derivatives. The disadvantage of this representation is that it is very difficult to construct the muscle length goals that will result in a coordinated movement such as walking. Despite this limitation, muscle space projection presents many attractive features. More DoFs permit coordination of different aspects of the movement (e.g., co-contraction) that are uncontrollable from a low-dimensional space. Furthermore, muscle projection provides a convenient representation for interfacing musculoskeletal and segmental level (proprioceptive) models and forms a basis for practical control applications, such as tendon-driven robots [4].

The various factors affecting kinematic and dynamic redundancy were identified and included in a closed form solution in order to calculate the feasible muscle forces. This not only demonstrated an effective approach for finding the family of feasible solutions, but also exposes the structure of the null space. The main advantage of the proposed approach is that the feasible muscle forces are action-specific, accounting for the dynamic evolution of the motion, while also satisfying the physiological constraints of the muscles, outlining the various factors that affect the solution space. The bottleneck of this method lies in the time complexity of the vertex enumeration algorithm, used for sampling the feasible space satisfying the constraints presented as linear inequalities. Given that the space defined by the inequality in Eq (32) is convex and bounded (Proposition 2), the complexity is cubic (*O*(*m*^3^)) with respect to the number of muscles. In highly complicated musculoskeletal models with many muscles, the aforementioned deterministic approach becomes computationally intractable due to the cubic time complexity growth. In such cases, randomized algorithms have to be employed to provide a representative sampling of the high-dimensional polytope [31].

Two case studies were presented to demonstrate the application of the proposed methods for calculating the feasible muscle forces in the context of an arbitrary task. In the first case study, a simplified arm model was used for simulating a hand movement. The muscle force variabilities and muscle group correlations were calculated. It was shown that 1) the variability of each muscle is inversely proportional to the importance of its contribution to the current movement and 2) there is a significant systematic correlation between different muscle pairs that emerges from the muscle routing properties and the functional constraints of the task. In the second case study, the feasible joint reaction loads were calculated for the hip joint during walking using a realistic full body model. From the resulting upper and lower bounds, it was concluded that misinterpretation of the results is possible if the null space solutions are ignored.

## Conclusion

In this study, we discuss several extensions of the kinematic and dynamic equations in order to characterize the redundant nature of musculoskeletal systems. The formal treatment of the problem with well-known techniques stemming from linear algebra and projection operators revealed the means of interaction between the different relations, providing hints towards addressing important scientific questions related to motor coordination. Forming a complete description enabled the development of powerful tools for investigating the feasible outcomes of the system as compared to the local techniques that are suitable for finding only a particular solution. The redundant nature of the musculoskeletal system introduces variability/uncertainty in simulated quantities leading to misinterpretation of the results if ignored. Therefore, this study provides the appropriate formalization to successfully address these issues, facilitating the application of broader types of studies in the realm of motor coordination.

## Appendices

### Projection operators

Let ***T***_***A***_ = ***AA***^+^ and ***F***_***A***_ = ***A***^+^ ***A*** be two operators derived from matrix ***A***, where ***A***^+^ is the MPP matrix. The following proofs rely on the Singular Value Decomposition (SVD) theorem (***A*** = ***U*Σ*V***^*T*^, ***A***^+^ = ***V*Σ**^+^***U***^*T*^) and the properties of the MPP (Definition 2).

(a) $T_{A}^{T}=T_{A}$ and $T_{A}^{2}=T_{A}$.
  $$\begin{array}{c} {\left(AA^{+}\right)}^{T}=AA^{+}\\ \left(AA^{+}\right)\left(AA^{+}\right)=A\left(A^{+}AA^{+}\right)=AA^{+} \end{array}$$
  where (***AA***^+^)^*T*^ = ***AA***^+^ and ***A***^+^ ***AA***^+^ = ***A***^+^ are properties of the MPP (Definition 2).
(b) $F_{A}^{T}=F_{A}$ and $F_{A}^{2}=F_{A}$. Similar to (a) with (***A***^+^ ***A***)^*T*^ = ***A***^+^ ***A*** and ***AA***^+^ ***A*** = ***A*** (Definition 2).
(c) ***T***_***A***_ ***A*** = ***AF***_***A***_ = ***A*** and ***A***^+^ ***T***_***A***_ = ***F***_***A***_ ***A***^+^ = ***A***^+^ can be proven using ***AA***^+^ ***A*** = ***A*** and ***A***^+^ ***AA***^+^ = ***A***^+^.
(d) ***T***_***A***_ is the projector onto the C(A).
  $$T_{A}=AA^{+}=U\Sigma V^{T}V\Sigma^{+}U^{T}=U\Sigma \Sigma^{+}U^{T}=\left[\begin{array}{ll} U_{r} & U_{n} \end{array}\right]\left[\begin{array}{cc} 1_{r} & 0\\ 0 & 0_{n} \end{array}\right]\left[\begin{array}{c} U_{r}^{T}\\ U_{n}^{T} \end{array}\right]=U_{r}U_{r}^{T}$$
  where ***V***^*T*^ ***V*** = ***I*** and *r* is the rank and *n* the nullity of ***A***.
(e) ***F***_***A***_ is the projector onto the R(A).
  $$F_{A}=A^{+}A=V\Sigma^{+}U^{T}U\Sigma V^{T}=V\Sigma^{+}\Sigma V^{T}=\left[\begin{array}{ll} V_{r} & V_{n} \end{array}\right]\left[\begin{array}{cc} 1_{r} & 0\\ 0 & 0_{n} \end{array}\right]\left[\begin{array}{c} V_{r}^{T}\\ V_{n}^{T} \end{array}\right]=V_{r}V_{r}^{T}$$
  where ***U***^*T*^ ***U*** = ***I***.
(f) $N_{T_{A}}=\left(I-T_{A}\right)$ projects onto the $N(A^{T})$, because C(A) and $N(A^{T})$ are mutually orthogonal complement (Eq (6)).
(g) $N_{F_{A}}=\left(I-F_{A}\right)$ projects onto the N(A), because R(A) and N(A) are mutually orthogonal complement (Eq (6)).
(h) $N_{T_{A}}A=0$ and $AN_{F_{A}}=0$.
  $$\begin{array}{c} N_{T_{A}}A=A-AA^{+}A=0 \end{array}$$
  $$\begin{array}{c} AN_{F_{A}}=A-AA^{+}A=0 \end{array}$$

### Task motion—Force duality

Given that $J_{t}^{+}$ is the MPP matrix and the definition of SVD ($J_{t}=U\Sigma V^{T}$), the following hold true:

(a) $J_{t}^{T}=V\Sigma U^{T}$
(b) $J_{t}^{+}=V\Sigma^{+}U^{T}$

thus their projection operators span the same subspaces.

### Muscle space inertia mass matrix

(a) $\Lambda_{m}$ is symmetric ($\Lambda_{m}=\Lambda_{m}^{T}$) iff ***M*** is symmetric.
  $$\begin{array}{c} \Lambda_{m}^{T}={\left(RM^{-1}R^{T}\right)}^{+T}={\left(RM^{-T}R^{T}\right)}^{+}={\left(RM^{-1}R^{T}\right)}^{+}=\Lambda_{m},\;iff\;M=M^{T} \end{array}$$
(b) $\Lambda_{m}$ remains invariant under the projection operator ***T***_***R***_.
  $$\begin{array}{c} \Lambda_{m}T_{R}={\left(RM^{-1}R^{T}\right)}^{+}RR^{+}=R^{+T}M\underset{R^{+}}{\underset{︸}{R^{+}RR^{+}}}={\left(RM^{-1}R^{T}\right)}^{+}=\Lambda_{m} \end{array}$$
(c) $\Lambda_{m}\Lambda_{m}^{+}=T_{R}$.
  $$\begin{array}{c} \;\begin{array}{cc} \Lambda_{m}\Lambda_{m}^{+} & ={\left(RM^{-1}R^{T}\right)}^{+}\left(RM^{-1}R^{T}\right)=R^{+T}M\underset{I}{\underset{︸}{R^{+}R}}M^{-1}R^{T}\\ & =R^{+T}R^{T}={\left(RR^{+}\right)}^{T}=RR^{+}=T_{R} \end{array} \end{array}$$
(d) $\Lambda_{m}N_{T_{R}}=0$. This can be shown using (b).

### Feasible muscle force space

(a) Let’s prove that Eq (34) defines a convex set C. For any x,y∈C and λ ∈ [0, 1], let ψ=λx+(1-λ)y then
  $$\begin{array}{c} Z\psi =\lambda Zx+(1-\lambda )Zy≼\lambda \beta +(1-\lambda )\beta =\beta \end{array}$$
  which proves the convexity property.
(b) Let’s prove that Eq (34) is bounded when the inequality is feasible and ***Z*** has full column rank. From the definition, this inequality has always the following form
  $$\begin{array}{c} a≼N_{T_{R}}f_{m0}≼b. \end{array}$$ (41)
  where ***a*** an ***b*** are finite vectors in $R^{m}$. If the system of inequalities is feasible and $N_{T_{R}}$ has full column rank, then ***f***_*m*0_ is upper and lower bounded. Even if the problem is feasible, if $N_{T_{R}}$ is rank deficient, then $\exists i:f_{m0}^{i}\to \pm \infty$, thus the feasible space is not bounded. Note, however, that we can always reduce the null space projection to full column rank by eliminating linearly dependent columns in $N_{T_{R}}$.

## Supporting information

S1 File Supporting information related to this publication. Implementation details and a detailed derivation of the simplified arm model.
(PDF)

## References

[1] Anderson FC, Pandy MG. Static and dynamic optimization solutions for gait are practically equivalent. Journal of Biomechanics. 2001;34(2):153–161. 10.1016/S0021-9290(00)00155-X 11165278

[2] Le Cavorzin P, Carrault G, Chagneau F, Rochcongar P, Allain H. A computer model of rigidity and related motor dysfunction in Parkinson’s disease. Movement disorders: official journal of the Movement Disorder Society. 2003;18(11):1257–65. 10.1002/mds.10532 14639665

[3] Zhang D, Poignet P, Bo APL, Ang WT. Exploring peripheral mechanism of tremor on neuromusculoskeletal model: a general simulation study. IEEE Transactions on Biomedical Engineering. 2009;56(10):2359–69. 10.1109/TBME.2009.2023979 19535320

[4] Deshpande AD, Balasubramanian R, Ko J, Matsuoka Y. Acquiring variable moment arms for index finger using a robotic testbed. IEEE Transactions on Biomedical Engineering. 2010;57(8):2034–2044. 10.1109/TBME.2010.2048326 20442038

[5] Steele KM, Tresch MC, Perreault EJ. Consequences of biomechanically constrained tasks in the design and interpretation of synergy analyses. Journal of Neurophysiology. 2015;113(7):2102–13. 10.1152/jn.00769.2013 25589591 PMC4416545

[6] Kutch JJ, Valero-Cuevas FJ. Challenges and new approaches to proving the existence of muscle synergies of neural origin. PLoS Computational Biology. 2012;8(5):1–11. 10.1371/journal.pcbi.1002434 22570602 PMC3342930

[7] Inouye JM, Valero-Cuevas FJ. Muscle Synergies Heavily Influence the Neural Control of Arm Endpoint Stiffness and Energy Consumption. PLoS Computational Biology. 2016;12(2):1–24. 10.1371/journal.pcbi.1004737 PMC4750997 26867014

[8] Bernshtein NA. The co-ordination and regulation of movements. Oxford: Pergamon Press; 1967.

[9] Latash ML, Levin MF, Scholz JP, Schöner G. Motor Control Theories and Their Applications. Medicina (Kaunas). 2010;46(6):382–92. 10.3390/medicina46060054 20944446 PMC3017756

[10] Loeb GE. Learning from the spinal cord. Journal of Physiology. 2001;533(1):111–117. 10.1111/j.1469-7793.2001.0111b.x 11351019 PMC2278609

[11] Houk JC, Rymer WZ. Neural control of muscle length and tension In: Handbook of Physiology, The Nervous System, Motor Control. John Wiley & Sons, Inc.; 2011 p. 257–323.

[12] Stein RB. What muscle variable(s) does the nervous system control in limb movements?. vol. 5 Cambridge University Press; 1982.

[13] Feldman AG. Once More on the Equilibrium-Point Hypothesis (*λ* Model) for Motor Control. Journal of Motor Behavior. 1986;18(1):17–54. 10.1080/00222895.1986.10735369 15136283

[14] Latash ML, Scholz JP, Schoner G. Motor Control Strategies Revealed in the Structure of Motor Variability. Exercise and Sport Sciences Reviews. 2002;30(1):26–31. 10.1097/00003677-200201000-00006 11800496

[15] Valero-Cuevas FJ, Hoffmann H, Kurse MU, Kutch JJ, Theodorou EA. Computational Models for Neuromuscular Function. IEEE Rev Biomed Eng. 2009;2:110–135. 10.1109/RBME.2009.2034981 21687779 PMC3116649

[16] Valero-Cuevas FJ. A Mathematical Approach to the Mechanical Capabilities of Limbs and Fingers. Adv Exo Med Biol. 2009;629:619–633. 10.1007/978-0-387-77064-2_33 PMC2839389 19227524

[17] Razavian RS, Mehrabi N, McPhee J. A model-based approach to predict muscle synergies using optimization: application to feedback control. Frontiers in Computational Neuroscience. 2015;9(121):1–13. 26500530 10.3389/fncom.2015.00121 PMC4593861

[18] Kutch JJ, Valero-Cuevas FJ. Muscle redundancy does not imply robustness to muscle dysfunction. Journal of Biomechanics. 2011;44(7):1264–1270. 10.1016/j.jbiomech.2011.02.014 21420091 PMC3090003

[19] Khatib O. Inertial Properties in Robotic Manipulation: An Object-Level Framework. International Journal of Robotics Research. 1995;14(1):19–36. 10.1177/027836499501400103

[20] Fisher W, Mujtaba S. Hybrid Position/Force Control: A Correct Formulation. The International Journal of Robotics Research. 1991;11(4):299–311. 10.1177/027836499201100403

[21] Aghili F. A unified approach for inverse and direct dynamics of constrained multibody systems based on linear projection operator: Applications to control and simulation. IEEE Transactions on Robotics. 2005;21(5):834–849. 10.1109/TRO.2005.851380

[22] Khatib O, Demircan E, De Sapio V, Sentis L, Besier TF, Delp SL. Robotics-based synthesis of human motion. Journal of Physiology-Paris. 2009;103(3-5):211–219. 10.1016/j.jphysparis.2009.08.004 PMC2782476 19665552

[23] Stanev D, Moustakas K. Simulation of Constrained Musculoskeletal Systems in Task Space. IEEE Transactions on Biomedical Engineering. 2018;65(2):307–318. 10.1109/TBME.2017.2764630 29053446

[24] Baerlocher P. Inverse kinematics techniques of the interactive posture control of articulated figures. Lausanne: EPFL; 2001.

[25] Righetti L, Buchli J, Mistry M, Schaal S. Inverse Dynamics Control of Floating-Base Robots With External Contraints: an Unified View. In: 2011 IEEE International Conference on Robotics and Automation; 2011. p. 1085–1090.

[26] Sentis L. Synthesis and Control of Whole-Body Behaviors in Humanoid Systems. 7 Stanford University; 2007.

[27] Zajac FE. Muscle and Tendon: Properties, Models, Scaling and Application to Biomechanics and Motor Control. Critical Reviews in Biomedical Engineering. 1989;17(4):359–411. 2676342

[28] Thelen DG. Adjustment of Muscle Mechanics Model Parameters to Simulate Dynamic Contractions in Older Adults. Journal of Biomechanical Engineering. 2003;125(1):70–77. 10.1115/1.1531112 12661198

[29] Millard M, Uchida T, Seth A, Delp SL. Flexing computational muscle: modeling and simulation of musculotendon dynamics. Journal of Biomechanical Engineering. 2013;135(2):1–12. 10.1115/1.4023390 PMC3705831 23445050

[30] Avis D, Fukuda K. A Pivoting Algorithm for Convex Hulls and Vertex Enumeration of Arrangements and Polyhedra. Discrete Computational Geometry. 1992;8(1):295–313. 10.1007/BF02293050

[31] Vempala SS. Geometric Random Walks: A Survey. MSRI Combinatorial and Computational Geometry. 2005;52:573–612.

[32] Delp SL, Loan PJ, Hoy MG, Zajac FE, Topp EL, Rosen JM. An interactive graphics-based model of the lower extremity to study orthopedic surgical procedures. IEEE Transactions on Biomedical Engineering. 1990;37(8):757–767. 10.1109/10.102791 2210784

[33] Delp SL, Anderson FC, Arnold AS, Loan PL, Habib A, John CT, et al OpenSim: Open-Source Software to Create and Analyze Dynamic Simulations of Movement. IEEE Transactions on Biomedical Engineering. 2007;54(11):1940–1950. 10.1109/TBME.2007.901024 18018689

[34] Loeb GE, Levine WS, He BJ. Understanding sensorimotor feedback through optimal control. Cold Spring Harbor Symposia on Quantitative Biology. 1990;55(1):791–803. 10.1101/SQB.1990.055.01.074 2132855

[35] Mileusnic MP, Brown IE, Lan N, Loeb GE. Mathematical models of proprioceptors. I. Control and transduction in the muscle spindle. Journal of Neurophysiology. 2006;96(4):1772–88. 10.1152/jn.00868.2005 16672301

[36] Mileusnic MP, Loeb GE. Mathematical models of proprioceptors. II. Structure and function of the Golgi tendon organ. Journal of Neurophysiology. 2006;96(4):1789–802. 10.1152/jn.00869.2005 16672300

[37] Erdemir A, McLean SG, Herzog W, Van Den Bogert AJ. Model-based estimation of muscle forces exerted during movements. Clinical Biomechanics. 2007;22(2):131–154. 10.1016/j.clinbiomech.2006.09.005 17070969

[38] Thelen DG, Anderson FC. Using computed muscle control to generate forward dynamic simulations of human walking from experimental data. Journal of Biomechanics. 2006;39(6):1107–15. 10.1016/j.jbiomech.2005.02.010 16023125

