# Supplementary material for: Modeling musculoskeletal kinematic and dynamic redundancy using null space projection
Source: PLoS One. 2019 Jan 2;14(1):e0209171. doi: 10.1371/journal.pone.0209171 (PMC6314624; doi:10.1371/journal.pone.0209171)
Supplement: S1 File — Implementation details and a detailed derivation of the simplified arm model. (PDF) [file pone.0209171.s001.pdf]

Supporting information

S1 Fig presents an overview of the various relationships between task, joint and muscle space. Implementation details are provided in section "Implementation details" so that simulation results presented here can be reproduced by others. A detailed derivation of the simplified arm model used in this study is presented in section "Simplified arm model".

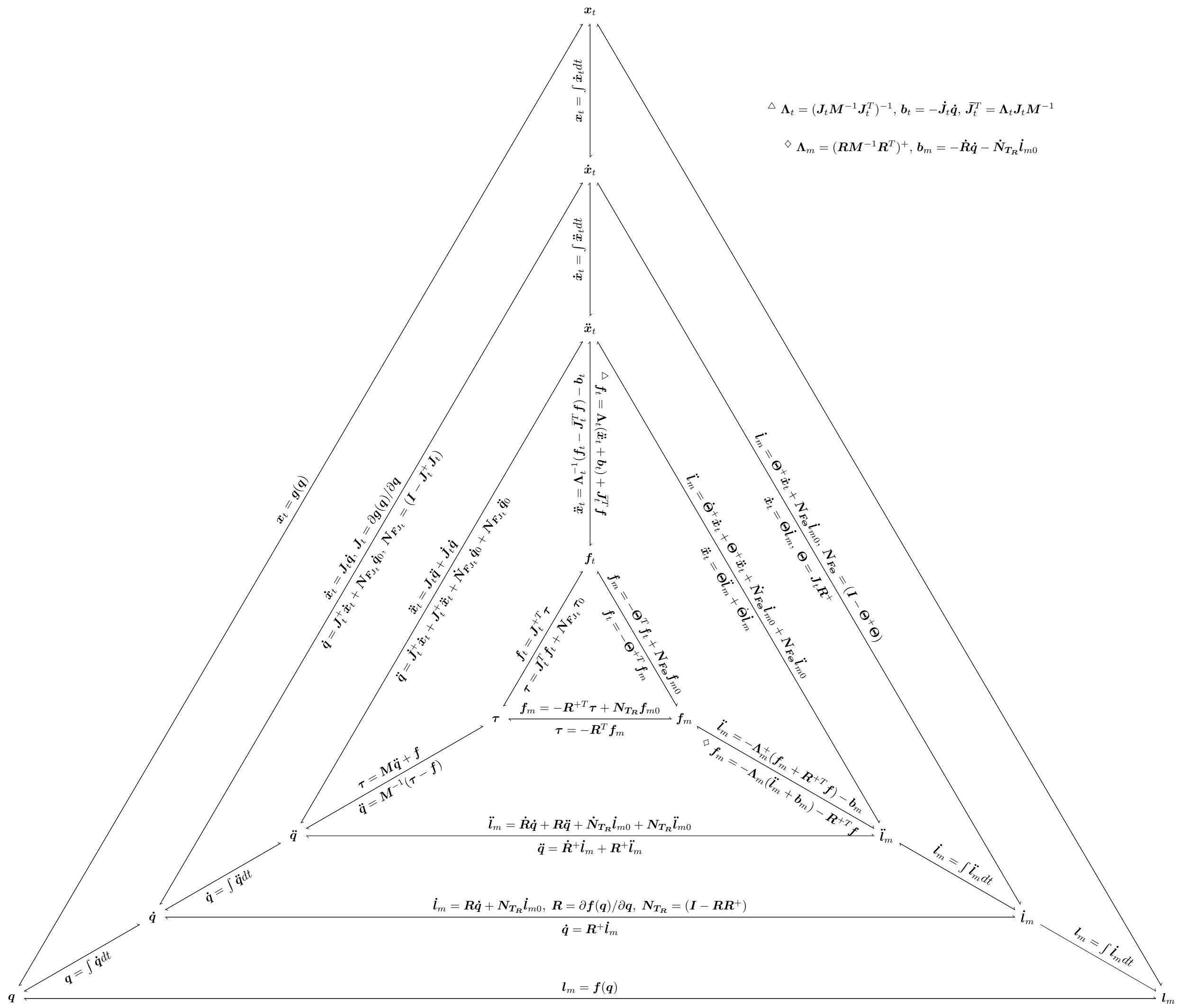

S1 Fig: Overview of various relationships between task, joint and muscle space.

Implementation details

The source code along with any related material for this publication are publicly available[1]. The following Python packages were utilized numpy, sympy, matplotlib and pandas for linear algebra manipulation, symbolic derivation, plotting and statistical analysis, respectively. The open-source OpenSim framework was used for modeling and simulation of musculoskeletal systems. A self-contained docker build file for an Ubuntu image, which contains the

[1] SimTK project: https://simtk.org/projects/redundancy
GitHub: https://github.com/mitkof6/musculoskeletal-redundancy

various libraries, dependencies and source code, was also prepared so that readers can inspect, run and reproduce the results without having to fetch or setup the necessary project dependencies.

Simplified arm model

The simplified arm model (S2 Fig) has three Degrees of Freedom (DoFs) and nine muscles, some of them being bi-articular. The analytical expressions of the Equations of Motion (EoMs) is given by

$$\boldsymbol{M}(\boldsymbol{q})\ddot{\boldsymbol{q}} + \boldsymbol{C}(\boldsymbol{q}, \dot{\boldsymbol{q}})\dot{\boldsymbol{q}} + \boldsymbol{\tau}_g(\boldsymbol{q}) = \boldsymbol{\tau} \tag{S1}$$

where $\boldsymbol{M} \in \Re^{n \times n}$ represents the inertia mass matrix, n the DoFs of the model, $\boldsymbol{q}, \dot{\boldsymbol{q}}, \ddot{\boldsymbol{q}} \in \Re^n$ the generalized coordinates and their derivatives, $\boldsymbol{C} \in \Re^{n \times n}$ the Coriolis and centrifugal matrix, $\boldsymbol{\tau}_g \in \Re^n$ the gravity contribution and $\boldsymbol{\tau}$ the specified generalized forces.

As the model is an open kinematic chain a simple procedure to derive the EoMs can be followed. Assuming that the spatial velocity (translational, rotational) of each body segment is given by $\boldsymbol{v}_b = [\boldsymbol{u}, \boldsymbol{\omega}]^T \in \Re^{6 \times 1}$, the Kinetic Energy (KE) of the system in body local coordinates is defined as

$$K = \frac{1}{2}\sum_{i=1}^{n_b}(m_i \boldsymbol{u}_i^2 + \boldsymbol{I}_i \boldsymbol{\omega}_i^2) = \frac{1}{2}\sum_{i=1}^{n_b} \boldsymbol{v}_i^T \boldsymbol{M}_i \boldsymbol{v}_i \tag{S2}$$

where $\boldsymbol{M}_i = diag(m_i, m_i, m_i, [\boldsymbol{I}_i]_{3\times3}) \in \Re^{6\times6}$ denotes the spatial inertia mass matrix, m_i the mass and $\boldsymbol{I}_i \in \Re^{3\times3}$ the inertia matrix of body i. The spatial quantities are related to the generalized coordinates by the body Jacobian $\boldsymbol{v}_b = \boldsymbol{J}_b\dot{\boldsymbol{q}},\ \boldsymbol{J}_b \in \Re^{6\times n}$. The total KE is coordinate invariant, thus it can be expressed in a different coordinate system

$$K = \frac{1}{2}\sum_{i=1}^{n_b} \boldsymbol{q}^T \boldsymbol{J}_i^T \boldsymbol{M}_i \boldsymbol{J}_i \boldsymbol{q}. \tag{S3}$$

Following the above definition, the inertia mass matrix of the system can be written as

$$\boldsymbol{M}(\boldsymbol{q}) = \sum_{i=1}^{n_b} \boldsymbol{J}_i^T \boldsymbol{M}_i \boldsymbol{J}_i. \tag{S4}$$

Furthermore, the Coriolis and centrifugal forces $\boldsymbol{C}(\boldsymbol{q}, \dot{\boldsymbol{q}})\dot{\boldsymbol{q}}$ can be determined directly from the inertia mass matrix

$$\boldsymbol{C}_{ij}(\boldsymbol{q},\ \dot{\boldsymbol{q}}) = \sum_{k=1}^{n} \boldsymbol{\Gamma}_{ijk}\ \dot{q}_k,\ i,\ j \in [1, \dots n],\ \boldsymbol{\Gamma}_{ijk} = \frac{1}{2}(\frac{\partial \boldsymbol{M}_{ij}(\boldsymbol{q})}{\partial q_k} + \frac{\partial \boldsymbol{M}_{ik}(\boldsymbol{q})}{\partial q_j} - \frac{\partial \boldsymbol{M}_{kj}(\boldsymbol{q})}{\partial q_i}) \tag{S5}$$

where the functions $\boldsymbol{\Gamma}_{ijk}$ are called the Christoffel symbols. The gravity contribution can be determined from the Potential Energy (PE) function

$$\boldsymbol{\tau}_g(\boldsymbol{q}) = \frac{\partial V(\boldsymbol{q})}{\partial \boldsymbol{q}},\ V(\boldsymbol{q}) = \sum_{i=1}^{n_b} m_i g h_i(\boldsymbol{q}) \tag{S6}$$

where $h_i(\boldsymbol{q})$ denotes the vertical displacement of body i with respect to the ground. In this derivation we chose to collect all forces that act on the system in the term $\boldsymbol{f}(\boldsymbol{q}, \dot{\boldsymbol{q}})$.

The muscle forces $\boldsymbol{f}_m$ are transformed into joint space generalized forces ($\boldsymbol{\tau}$) by the moment arm matrix ($\boldsymbol{\tau} = -\boldsymbol{R}^T \boldsymbol{f}_m$). For a n-lateral polygon it can be shown that the derivative of the side length with respect to the opposite angle is the moment arm component. As a consequence, when expressing the muscle length as a function of the generalized coordinates of the model, the moment arm matrix is evaluated by $\boldsymbol{R} = \partial \boldsymbol{l}_m(\boldsymbol{q})/\partial \boldsymbol{q}$. The analytical expressions of the EoMs following our convention are provided below (Eq. (S7)). The mass properties of the model are summarized in S1 Table and the muscle parameters in S2 Table.

$$\begin{aligned} \boldsymbol{M}(\boldsymbol{q})\ddot{\boldsymbol{q}} + \boldsymbol{f}(\boldsymbol{q}, \dot{\boldsymbol{q}}) &= \boldsymbol{\tau} \\ \boldsymbol{\tau} &= -\boldsymbol{R}^T(\boldsymbol{q})\boldsymbol{f}_m \end{aligned} \tag{S7}$$

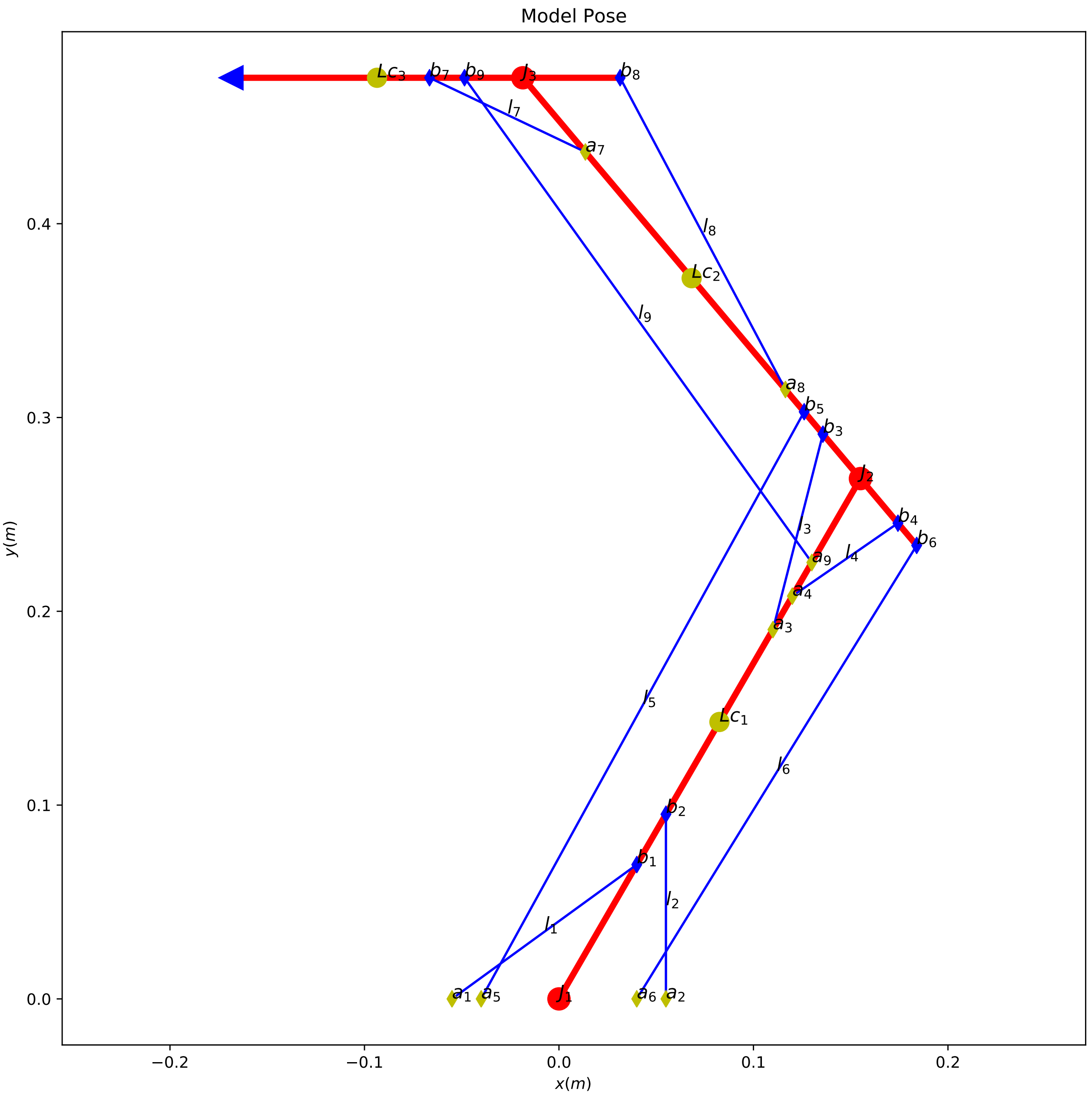

S2 Fig: Diagram of the simplified arm model. The model has three DoFs and nine muscles, some of them being bi-articular. The muscle origins are labeled as a_i and the muscle insertions as b_i. l_i stands for muscle length, Lc_i for center of mass and J_i for joint center.

$$\begin{aligned} M_{1,1} = {} & Iz_1 + Iz_2 + Iz_3 + L_1^2 m_2 + L_1^2 m_3 + 2L_1 L_2 m_3 \cos(q_2(t)) + 2L_1 Lc_2 m_2 \cos(q_2(t)) \\ & + 2L_1 Lc_3 m_3 \cos(q_2(t) + q_3(t)) + L_2^2 m_3 + 2L_2 Lc_3 m_3 \cos(q_3(t)) + Lc_1^2 m_1 + Lc_2^2 m_2 + Lc_3^2 m_3 \end{aligned} \tag{S8}$$

$$\begin{aligned} M_{1,2} = {} & Iz_2 + Iz_3 + L_1 L_2 m_3 \cos(q_2(t)) + L_1 Lc_2 m_2 \cos(q_2(t)) + L_1 Lc_3 m_3 \cos(q_2(t) + q_3(t)) \\ & + L_2^2 m_3 + 2L_2 Lc_3 m_3 \cos(q_3(t)) + Lc_2^2 m_2 + Lc_3^2 m_3 \end{aligned} \tag{S9}$$

$$M_{1,3} = Iz_3 + L_1 Lc_3 m_3 \cos(q_2(t) + q_3(t)) + L_2 Lc_3 m_3 \cos(q_3(t)) + Lc_3^2 m_3 \tag{S10}$$

$$
\begin{aligned}
M_{2,1} = {} & Iz_2 + Iz_3 + L_1 L_2 m_3 \cos\left(q_2(t)\right) + L_1 Lc_2 m_2 \cos\left(q_2(t)\right) + L_1 Lc_3 m_3 \cos\left(q_2(t) + q_3(t)\right) \\
& + L_2^2 m_3 + 2 L_2 Lc_3 m_3 \cos\left(q_3(t)\right) + Lc_2^2 m_2 + Lc_3^2 m_3
\end{aligned} \tag{S11}
$$

$$
M_{2,2} = Iz_2 + Iz_3 + L_2^2 m_3 + 2 L_2 Lc_3 m_3 \cos\left(q_3(t)\right) + Lc_2^2 m_2 + Lc_3^2 m_3 \tag{S12}
$$

$$
M_{2,3} = Iz_3 + L_2 Lc_3 m_3 \cos\left(q_3(t)\right) + Lc_3^2 m_3 \tag{S13}
$$

$$
M_{3,1} = Iz_3 + L_1 Lc_3 m_3 \cos\left(q_2(t) + q_3(t)\right) + L_2 Lc_3 m_3 \cos\left(q_3(t)\right) + Lc_3^2 m_3 \tag{S14}
$$

$$
M_{3,2} = Iz_3 + L_2 Lc_3 m_3 \cos\left(q_3(t)\right) + Lc_3^2 m_3 \tag{S15}
$$

$$
M_{3,3} = Iz_3 + Lc_3^2 m_3 \tag{S16}
$$

$$
\begin{aligned}
f_1 = {} & Lc_1 g m_1 \cos\left(q_1(t)\right) + g m_2 \left(L_1 \cos\left(q_1(t)\right) + Lc_2 \cos\left(q_1(t) + q_2(t)\right)\right) \\
& + g m_3 \left(L_1 \cos\left(q_1(t)\right) + L_2 \cos\left(q_1(t) + q_2(t)\right) + Lc_3 \cos\left(q_1(t) + q_2(t) + q_3(t)\right)\right) \\
& - 2 L_1 L_2 m_3 \sin\left(q_2(t)\right) \frac{d}{dt} q_1(t) \frac{d}{dt} q_2(t) + L_1 L_2 m_3 \sin\left(q_2(t)\right) \left(\frac{d}{dt} q_2(t)\right)^2 \\
& + 2 L_1 Lc_2 m_2 \sin\left(q_2(t)\right) \frac{d}{dt} q_1(t) \frac{d}{dt} q_2(t) + L_1 Lc_2 m_2 \sin\left(q_2(t)\right) \left(\frac{d}{dt} q_2(t)\right)^2 \\
& + 2 L_1 Lc_3 m_3 \sin\left(q_2(t) + q_3(t)\right) \frac{d}{dt} q_1(t) \frac{d}{dt} q_2(t) + 2 L_1 Lc_3 m_3 \sin\left(q_2(t) + q_3(t)\right) \frac{d}{dt} q_1(t) \frac{d}{dt} q_3(t) \\
& + L_1 Lc_3 m_3 \sin\left(q_2(t) + q_3(t)\right) \left(\frac{d}{dt} q_2(t)\right)^2 + 2 L_1 Lc_3 m_3 \sin\left(q_2(t) + q_3(t)\right) \frac{d}{dt} q_2(t) \frac{d}{dt} q_3(t) \\
& + L_1 Lc_3 m_3 \sin\left(q_2(t) + q_3(t)\right) \left(\frac{d}{dt} q_3(t)\right)^2 + 2 L_2 Lc_3 m_3 \sin\left(q_3(t)\right) \frac{d}{dt} q_1(t) \frac{d}{dt} q_3(t) \\
& + 2 L_2 Lc_3 m_3 \sin\left(q_3(t)\right) \frac{d}{dt} q_2(t) \frac{d}{dt} q_3(t) + L_2 Lc_3 m_3 \sin\left(q_3(t)\right) \left(\frac{d}{dt} q_3(t)\right)^2
\end{aligned} \tag{S17}
$$

$$
\begin{aligned}
f_2 = {} & -L_2 Lc_3 m_3 \left(\frac{d}{dt} q_1(t) + \frac{d}{dt} q_2(t) + \frac{d}{dt} q_3(t)\right) \sin\left(q_3(t)\right) \frac{d}{dt} q_3(t) - L_2 Lc_3 m_3 \sin\left(q_3(t)\right) \frac{d}{dt} q_2(t) \frac{d}{dt} q_3(t) \\
& + Lc_2 g m_2 \cos\left(q_1(t) + q_2(t)\right) + g m_3 \left(L_2 \cos\left(q_1(t) + q_2(t)\right) + Lc_3 \cos\left(q_1(t) + q_2(t) + q_3(t)\right)\right) \\
& + \Bigg(L_1 \left(L_2 m_3 \sin\left(q_2(t)\right) + Lc_2 m_2 \sin\left(q_2(t)\right) + Lc_3 m_3 \sin\left(q_2(t) + q_3(t)\right)\right) \frac{d}{dt} q_1(t) \\
& \qquad - L_2 Lc_3 m_3 \sin\left(q_3(t)\right) \frac{d}{dt} q_3(t)\Bigg) \frac{d}{dt} q_1(t)
\end{aligned} \tag{S18}
$$

$$
\begin{aligned}
f_3 = {} & Lc_3 g m_3 \cos\left(q_1(t) + q_2(t) + q_3(t)\right) + Lc_3 m_3 \Bigg(L_1 \sin\left(q_2(t) + q_3(t)\right) \left(\frac{d}{dt} q_1(t)\right)^2 + L_2 \sin\left(q_3(t)\right) \left(\frac{d}{dt} q_1(t)\right)^2 \\
& + 2 L_2 \sin\left(q_3(t)\right) \frac{d}{dt} q_1(t) \frac{d}{dt} q_2(t) + L_2 \sin\left(q_3(t)\right) \left(\frac{d}{dt} q_2(t)\right)^2\Bigg)
\end{aligned} \tag{S19}
$$

$$
R_{1,1} = -\frac{a_1 b_1 \sin\left(q_1(t)\right)}{\sqrt{a_1^2 + 2 a_1 b_1 \cos\left(q_1(t)\right) + b_1^2}} \tag{S20}
$$

$$
R_{1,2} = 0 \tag{S21}
$$

$$
R_{1,3} = 0 \tag{S22}
$$

$$
R_{2,1} = \frac{a_2 b_2 \sin\left(q_1(t)\right)}{\sqrt{a_2^2 - 2 a_2 b_2 \cos\left(q_1(t)\right) + b_2^2}} \tag{S23}
$$

$$
R_{2,2} = 0 \tag{S24}
$$

$$
R_{2,3} = 0 \tag{S25}
$$

$$
R_{3,1} = 0 \tag{S26}
$$

$$R_{3,2} = -\frac{b_3\left(2L_1 - 2a_3\right)\sin\left(q_2(t)\right)}{2\sqrt{b_3^2 + b_3\left(2L_1 - 2a_3\right)\cos\left(q_2(t)\right) + \left(L_1 - a_3\right)^2}} \tag{S27}$$

$$R_{3,3} = 0 \tag{S28}$$

$$R_{4,1} = 0 \tag{S29}$$

$$R_{4,2} = \frac{b_4\left(2L_1 - 2a_4\right)\sin\left(q_2(t)\right)}{2\sqrt{b_4^2 - b_4\left(2L_1 - 2a_4\right)\cos\left(q_2(t)\right) + \left(L_1 - a_4\right)^2}} \tag{S30}$$

$$R_{4,3} = 0 \tag{S31}$$

$$R_{5,1} = -\frac{a_5\left(L_1\sin\left(q_1(t)\right) + b_5\sin\left(q_1(t) + q_2(t)\right)\right)}{\sqrt{L_1^2 + 2L_1a_5\cos\left(q_1(t)\right) + 2L_1b_5\cos\left(q_2(t)\right) + a_5^2 + 2a_5b_5\cos\left(q_1(t) + q_2(t)\right) + b_5^2}} \tag{S32}$$

$$R_{5,2} = -\frac{b_5\left(L_1\sin\left(q_2(t)\right) + a_5\sin\left(q_1(t) + q_2(t)\right)\right)}{\sqrt{L_1^2 + 2L_1a_5\cos\left(q_1(t)\right) + 2L_1b_5\cos\left(q_2(t)\right) + a_5^2 + 2a_5b_5\cos\left(q_1(t) + q_2(t)\right) + b_5^2}} \tag{S33}$$

$$R_{5,3} = 0 \tag{S34}$$

$$R_{6,1} = \frac{a_6\left(L_1\sin\left(q_1(t)\right) - b_6\sin\left(q_1(t) + q_2(t)\right)\right)}{\sqrt{L_1^2 - 2L_1a_6\cos\left(q_1(t)\right) - 2L_1b_6\cos\left(q_2(t)\right) + a_6^2 + 2a_6b_6\cos\left(q_1(t) + q_2(t)\right) + b_6^2}} \tag{S35}$$

$$R_{6,2} = \frac{b_6\left(L_1\sin\left(q_2(t)\right) - a_6\sin\left(q_1(t) + q_2(t)\right)\right)}{\sqrt{L_1^2 - 2L_1a_6\cos\left(q_1(t)\right) - 2L_1b_6\cos\left(q_2(t)\right) + a_6^2 + 2a_6b_6\cos\left(q_1(t) + q_2(t)\right) + b_6^2}} \tag{S36}$$

$$R_{6,3} = 0 \tag{S37}$$

$$R_{7,1} = 0 \tag{S38}$$

$$R_{7,2} = 0 \tag{S39}$$

$$R_{7,3} = -\frac{b_7\left(2L_2 - 2a_7\right)\sin\left(q_3(t)\right)}{2\sqrt{b_7^2 + b_7\left(2L_2 - 2a_7\right)\cos\left(q_3(t)\right) + \left(L_2 - a_7\right)^2}} \tag{S40}$$

$$R_{8,1} = 0 \tag{S41}$$

$$R_{8,2} = 0 \tag{S42}$$

$$R_{8,3} = \frac{b_8\left(2L_2 - 2a_8\right)\sin\left(q_3(t)\right)}{2\sqrt{b_8^2 - b_8\left(2L_2 - 2a_8\right)\cos\left(q_3(t)\right) + \left(L_2 - a_8\right)^2}} \tag{S43}$$

$$R_{9,1} = 0 \tag{S44}$$

$$R_{9,2} = \frac{-\frac{L_2}{2}\left(2L_1 - 2a_9\right)\sin\left(q_2(t)\right) - \frac{b_9}{2}\left(2L_1 - 2a_9\right)\sin\left(q_2(t) + q_3(t)\right)}{\sqrt{L_2^2 + 2L_2b_9\cos\left(q_3(t)\right) + L_2\left(2L_1 - 2a_9\right)\cos\left(q_2(t)\right) + b_9^2 + b_9\left(2L_1 - 2a_9\right)\cos\left(q_2(t) + q_3(t)\right) + \left(L_1 - a_9\right)^2}} \tag{S45}$$

$$R_{9,3} = \frac{-L_2b_9\sin\left(q_3(t)\right) - \frac{b_9}{2}\left(2L_1 - 2a_9\right)\sin\left(q_2(t) + q_3(t)\right)}{\sqrt{L_2^2 + 2L_2b_9\cos\left(q_3(t)\right) + L_2\left(2L_1 - 2a_9\right)\cos\left(q_2(t)\right) + b_9^2 + b_9\left(2L_1 - 2a_9\right)\cos\left(q_2(t) + q_3(t)\right) + \left(L_1 - a_9\right)^2}} \tag{S46}$$

S1 Table: Body parameters: mass, inertia, length and center of mass (CoM).

**Bodies**	$\boldsymbol{m\ (kg)}$	$\boldsymbol{I_z\ (kg \cdot m^2)}$	$\boldsymbol{L\ (m)}$	$\boldsymbol{CoM\ (m)}$
arm	1.93	0.0141	0.31	0.165
forearm	1.32	0.0120	0.27	0.135
hand	0.35	0.0010	0.15	0.075

S2 Table: Muscle parameters: muscle origin, insertion and maximum force.

**Muscle**	a (m)	b (m)	$f_{\max}$ (N)
m_1	0.055	0.080	50
m_2	0.055	0.110	50
m_3	0.220	0.030	50
m_4	0.240	0.030	50
m_5	0.040	0.045	25
m_6	0.040	0.045	25
m_7	0.220	0.048	50
m_8	0.060	0.050	50
m_9	0.260	0.030	25
